# LFQ-Based Peptide and Protein Intensity Differential Expression Analysis

**DOI:** 10.1021/acs.jproteome.2c00812

**Published:** 2023-05-23

**Authors:** Mingze Bai, Jingwen Deng, Chengxin Dai, Julianus Pfeuffer, Timo Sachsenberg, Yasset Perez-Riverol

**Affiliations:** † Chongqing Key Laboratory of Big Data for Bio Intelligence, Chongqing University of Posts and Telecommunications, Chongqing 400065, China; ‡ State Key Laboratory of Proteomics, Beijing Proteome Research Center, National Center for Protein Sciences (Beijing), Beijing Institute of Life Omics, Beijing 102206, China; § Algorithmic Bioinformatics, Freie Universität Berlin, Berlin 14195, Germany; ∥ Visualization and Data Analysis, Zuse Institute Berlin, Berlin 14195, Germany; ⊥ Institute for Bioinformatics and Medical Informatics, University of Tübingen, Tübingen 72076, Germany; # European Molecular Biology Laboratory, European Bioinformatics Institute (EMBL-EBI), Wellcome Trust Genome Campus, Hixton, Cambridge CB10 1SD, United Kingdom

**Keywords:** data analysis, imputation and normalization algorithms, mass spectrometry proteomics, protein expression, protein fold changes

## Abstract

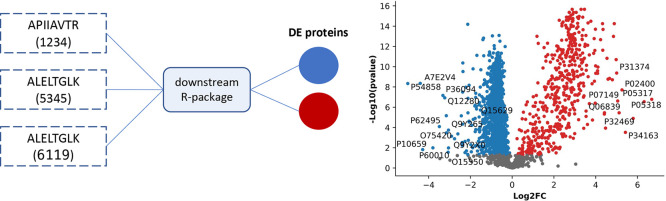

Testing for significant differences in quantities at the protein level is a common goal of many LFQ-based mass spectrometry proteomics experiments. Starting from a table of protein and/or peptide quantities from a given proteomics quantification software, many tools and R packages exist to perform the final tasks of imputation, summarization, normalization, and statistical testing. To evaluate the effects of packages and settings in their substeps on the final list of significant proteins, we studied several packages on three public data sets with known expected protein fold changes. We found that the results between packages and even across different parameters of the same package can vary significantly. In addition to usability aspects and feature/compatibility lists of different packages, this paper highlights sensitivity and specificity trade-offs that come with specific packages and settings.

## 1 Introduction

Proteomics has become a key technology to understand and characterize protein expression,^1,2^ interactions, and sequence modifications^3^ in state-of-the-art biology research.^2^ Quantitative bottom-up proteomics has been dominated by three different approaches: in vivo metabolic labeling,^4^ in vitro labeling,^5^ and label-free methods.^6^ In quantitative label-free, no isotopes or labels are added to the sample and the samples are not multiplexed in the same runs. Label-free approaches typically require fewer sample experimental steps, and differential expression analysis can simultaneously be performed across many samples.^7^

From the bioinformatics data analysis perspective, label-free methods and labeled experiments share multiple steps including mass spectra preprocessing, peptide identification, and protein inference.^8^ For peptide/protein identification tasks, multiple bioinformatics tools are available such as MaxQuant,^9^ MS-GF+,^10^ and PeptideShaker^11^ as well as cloud-based workflows like quantms^12^ or Galaxy proteomics.^13,14^ However, the quantification step is significantly different, including multiple substeps such as feature retention time alignment and feature detection.^8,14,15^ One of the main challenges in label-free based experiments is the high number of missing values across samples and replicates, which makes other substeps like protein expression normalization difficult.^15,16^ The high number of missing values presented in LFQ-based experiments has triggered the development of multiple R-packages including different algorithms for intensity normalization and imputation.

In this work, we studied multiple R-packages that enable the normalization, imputation, and differential expression analysis on LFQ-based intensity proteomics experiments. Previous works have mainly focused on evaluating the software that performs peptide identification, protein inference,^17^ and the generation of protein intensity tables.^8,18,19^ We first briefly describe the main packages and tools that enable the statistical analysis of LFQ data sets from peptide or protein intensity data. While multiple packages and tools are available for statistical analysis of these data, we selected some of the most relevant ones and novel implementations including MSstats,^20^ Perseus,^21^ Proteus,^22^ prolfqua,^23^ ProVision,^24^ LFQ-Analyst,^25^ Eatomics,^26^ ProStaR,^27^ and msqrob2.^28−30^ Finally, we used three different data sets—UPS spiked data set,^31^ large-scale mix data set^32^ and toxicology data set^33^—to evaluate the performance of each tool and discuss some of the advantages and disadvantages of their use with different types of data sets.

## 2 R-Packages for Statistical Analysis of Quantitative LFQ-Based Data

Statistical validation and assessment of protein expression data have been dominated by the R language. R (https://www.r-project.org/) is a popular framework for statistics and machine learning analysis. Many R packages are developed for bioinformatics analysis and visualization, especially due to the rapid increase of libraries provided by Bioconductor.^34^ In 2014, Gatto et al.^35^ showed that R and Bioconductor are the perfect environments for statistical analysis of proteomics data and indispensable for computational proteomics research. In addition to R-packages, R-Shiny applications are commonly used in proteomics not only to perform the DE analysis but also to interactively explore the results.^36^ R-Shiny is an R framework to build interactive web apps including dashboards and interactive plots (https://shiny.rstudio.com/).

Table 1 presents a group of recently published or commonly used R-packages and R-Shiny tools for statistical analysis of quantitative LFQ-based data. More details on imputation and normalization methods can be found in Supplementary Table 1, including the description of each method, and the default methods for each tool. We selected the packages based on the number of uses in PubMed, the novelty of the algorithms and methods employed for the statistical analysis, the maintainability, and the user interface. We will not study independent scripts or adaptions of other omics packages to the field of proteomics. We focused the study on the imputation and normalization algorithms of each tool, other parts of the algorithms such as multiple testing correction are not explored because most of these tools use Benjamini-Hochberg by default.^37^Supplementary Tables 2 and 3 show the required parameters to run each tool and the quality metrics computed by each tool, respectively.

**Table 1:** Comparison of the Nine Evaluated Packages and Tools in Different Metrics and Properties^a^

	Open source	It has been benchmarked	QC reports	Input intensity data type	Normalization method(s)	Imputation method(s)
MSstats	√	√	√	Pep	Equalize median, Quantile, Global standards	Accelerated failure time model, cutoffCensore, censoredInt, MaxQuantileforCensored
msqrob2	√	√	×	Pep	quantiles, quantiles.robust, vsn, center.median, center.mean, max, sum, div.mean, div.median, diff.median	×
ProStaR	√	√	×	Pep/Prot	Global Quantile alignment and Centering, Column sums, vsn, LOESS	Det quantile, slsa, KNN, fixed value
Proteus	√	√	×	Pep/Prot	Equalize median, Quantile	Mean-variance (by limma package)
prolfqua	√	√	√	Pep/Prot	robust scale	Group mean imputation
ProVision	√	×	×	Prot	×	Normal distribution like Perseus
LFQ-Analyst	√	√	√	Prot	×	Normal distribution like Perseus, KNN, bpca, QRILC, MLE, MinDet, MinProb, min, zero
Eatomics	√	×	√	Prot	VSN	Normal distribution like Perseus, KNN, MinDet, QRILC
Perseus	×	√	√	Prot	Z-score, remove batch effect, Un-Z-score	A down-shifted Gaussian distribution, constant, ImputeLCMD

a The input intensity data type refers to the intensity data supported by the package: (Pep) peptide intensity; (Prot) protein intensity.

### 2.1 MSstats

MSstats^20^ (https://github.com/Vitek-Lab/MSstats) is an open-source R-package for peptide and protein quantification in mass spectrometry-based proteomics. MSstats supports multiple data acquisition types: data-dependent acquisition (DDA), both LFQ and label-based workflows, data-independent acquisition (DIA), and targeted approaches. It uses a set of flexible linear mixed models to summarize the protein or peptide abundance in a single biological replicate or condition and perform the relative quantification of proteins across conditions. A simple tabular file containing the intensity of the identified peptides and proteins is needed as input. MSstats provides multiple adapters to transform the output of proteomics analysis tools like MaxQuant, Skyline^38^ or OpenMS into MSstats input format. Remarkably, MSstats is one of the most documented and actively maintained tools for differential expression analysis.

### 2.2 Proteus and limma

Proteus^22^ (https://github.com/bartongroup/Proteus) is used for DE analysis of MaxQuant output data, and differential expression analysis based on the popular algorithm/package limma.^39^ Proteus supports two normalization methods: equalize median and quantile, and it uses a mean-variance relationship to estimate variance (limma) where data are missing. The Proteus Shiny application allows users to perform the analysis with one click if the data is provided in MaxQuant format. Shiny-based volcano plots and fold-change intensity plots enable users to interact with differentially expressed proteins of interest and to view the significant results in detail. One of the other advantages of Proteus is that it has a lot of default data processing parameters that enable nonexpert users to perform a robust analysis of their data without the need to try multiple combinations of parameters. However, no quality control (QC) report is provided by the package making it difficult to assess the quality of the data and reproducibility of the experiment.

### 2.3 prolfqua

prolfqua^23^ (https://github.com/fgcz/prolfqua) integrates the basic steps of a differential expression analysis workflow: quality control, data normalization, protein aggregation, statistical modeling, hypothesis testing, and sample size estimation. The modular design of prolfqua enables users to select the optimal differential expression analysis algorithm. prolfqua supports four normalization methods: quantile, variance stabilizing normalization (vsn),^40^ log2 transform, and z-scale, and it enables imputation by a group-mean model. It provides a set of reports for users such as peptide intensity variance across samples, a scatterplot matrix of intensity correlation across samples and replicates, and a heatmap of missing values clustered by samples (example report https://fgcz.github.io/prolfqua/articles/QCandSampleSize.html). prolfqua supports the output of multiple tools including MaxQuant,^9^ Skyline,^38^ DIA-NN,^41^ and MSstats.^20^

### 2.4 ProVision

ProVision^24^ (https://github.com/JamesGallant/ProVision) is an R-shiny web application to facilitate the analysis of LFQ and TMT proteomics experiments. ProVision is designed for end-users (e.g., biologists), with a set of graphical interfaces to guide the users through data processing, parameter selection, and result presentation. In addition, it provides several parameters for users to interact with important filtering and statistical processes. ProVision only supports the MaxQuant output format as input and requires users to manually annotate the experimental design. In addition, the tool lacks QC reports that enable understanding and assessing the quality of the data.

### 2.5 LFQ-Analyst

LFQ-Analyst^25^ (https://github.com/MonashBioinformaticsPlatform/LFQ-Analyst) is an interactive, R-Shiny-based platform for quickly and easily analyzing and visualizing unlabeled proteomics data preprocessed with MaxQuant. LFQ-Analyst can process LFQ intensity, and its quality control report contains multiple visualization plots (volcano plots, heatmaps, and box plots) of differentially expressed. However, LFQ-Analyst has fewer statistical parameters for users to choose from, supports only MaxQuant format files and needs users to manually annotate experimental design files before the analysis.

### 2.6 Eatomics

Eatomics^26^ (https://github.com/Millchmaedchen/Eatomics) is also an R-shiny application for the interactive exploration of quantitative proteomics data from MaxQuant, integrating quality control, differential abundance analysis, and enrichment analysis. It has a variety of interactive exploration possibilities and a unique experimental design setup module that interactively transforms a given research hypothesis into a differential abundance and enrichment analysis formula. One advantage of Eatomics is that it has built-in detailed user tutorials to help users get started and can be run with one click after uploading the input file.

### 2.7 DAPAR and ProStaR

DAPAR and ProStaR^27^ (http://www.prostar-proteomics.org/) are two tools dedicated to the discovery of differential analysis of quantitative data generated by proteomic experiments. DAPAR is an R-package which provides five processing steps (filtering, normalization, imputation, aggregation, and difference analysis), based on those functions, ProStaR provides an R-Shiny web platform for interactive exploring. The advantage of ProStaR lies in its ability to do online analysis and embed detailed user tutorials to help users get started. There are various types of inputs, such as proteinGroups.txt from MaxQuant, Proline^42^ and MSnset^43^ files. Users need to manually annotate the condition and select multiple parameters, and they do not provide QC reports on the analysis results or the original intensity data.

### 2.8 msqrob2

msqrob2^28−30^ (https://github.com/statOmics/msqrob2/) is a free and open-source R package that can handle virtually any experimental proteomics design. msqrob supports multiple types of inputs, including MaxQuant,^9^ moFF,^44^ and mzTab.^45,46^ msqrob2 can use both R script and Shiny application (https://github.com/statOmics/msqrob2gui) for analysis and has detailed instruction manuals and videos to help new users get started. However, no matter which method is used, users need to manually annotate the condition and cannot obtain the QC report.

### 2.9 Perseus

In addition to the open-source R packages and R-Shiny applications, we explore the performance of the popular tool Perseus for users familiar with MaxQuant. Perseus^21^ (https://maxquant.net/perseus/) is designed for DE analysis of quantitative results in the MaxQuant ecosystem. Perseus is a desktop application which offers a wide variety of algorithms for MaxQuant data normalization, imputation, batch correction and differential expression analysis. Users need to manually annotate the condition during data processing and can choose different types of intensity: raw intensities, LFQ intensity or IBAQ values. It provides quality control reports but requires users to generate them manually. The tool provides an extensive number of supporting materials and online tutorials (https://www.youtube.com/@MaxQuantChannel).

## 3 Evaluation Framework

### 3.1 Benchmark Data Sets

Three data sets were used to evaluate each package. The LFQ-based data set PXD000279 (“UPS spiked dataset”),^31^ consists of two *E. coli* digested samples (with 4 replicates for each sample); each half of the samples are enriched with one of two “Universal Protein Standards” (UPS1 and UPS2). Both samples contained the same 48 recombinant human proteins, which were either mixed in equal amounts (UPS1) or spanned multiple orders of magnitude at a determined ratio (UPS2). Based on the experimental design, 40 of the 48 UPS proteins and none of the *E. coli* proteins should be detected as differentially expressed. This data set has been extensively used to evaluate LFQ-based differential expression tools^14,25,31^ and algorithms.^15^

In addition, two other more complex data sets were used PXD007145^32^ (“large-scale mix dataset”) and PXD020248^33^ (“toxicology dataset”). The large-scale mix data set contains a multiple species mixture, in which Yeast proteome was diluted into fixed ratios of 1:4:10 and added to a background of 1:1:1 human proteome to simulate the real experimental data. Six technical replicates were used for each sample to measure the coefficient of variations. The toxicology data set is a cell line hepatocytes sample (HepG2) treated with benzo[*a*]pyrene (BaP) using a concentration of 2 μM. The original work^33^ benchmarked TMT and LFQ analytical methods using the same sample. In the present study, we used the LFQ part to benchmark the peptide intensity-based tools MSstats, Proteus, and msqrob2.

### 3.2 Peptide and Protein Quantitation Tools

#### MaxQuant

To evaluate each tool’s parameter combinations, algorithms for data processing and protein quantification analysis, we analyzed the data sets with MaxQuant.^9^ Raw data were processed with MaxQuant (version v1.6.10.43) before the DE analysis with each tool. We used default parameters except that “the min ratio of LFQ” was set as 1 and “matching between runs” was enabled. The results from MaxQuant analysis and the parameters file used can be downloaded from the following repository (https://github.com/ypriverol/quantms-research/tree/main/r-research).

#### quantms

quantms (and its predecessor proteomicsLFQ^12^) is a cloud-based workflow that uses OpenMS^47^ tools and DIA-NN^41^ to enable quantitative analysis of LFQ data-dependent (LFQ-DDA) and independent acquisition (LFQ-DIA) and TMT data (https://quantms.readthedocs.io/en/latest/). In the present work, we used the LFQ-DDA subworkflow of the pipeline on the three data sets to do the peptide quantification benchmarking. The subworkflow performs peptide identifications using Comet^48^ and MSGF+^10^ and feature detection using proteomicsLFQ in OpenMS.^47^

All the scripts and data used to generate the figures and tables of the paper can be found in the following repository (https://github.com/ypriverol/quantms-research/tree/main/r-research). The parameter selections of Shiny tools are shown in Supplementary Note 1.

## 4 Results and Discussion

### 4.1 Protein-Based Analysis

Table 2 shows the performance of each evaluated R-package with the UPS spiked and large-scale mix data sets. Both data sets were analyzed with MaxQuant, and protein intensity tables were used as the starting point of the DE analysis. For each protein in the UPS spiked data set, if both the true and estimated protein abundance ratios are greater than 1 in the same direction and the adjusted p-value is less than 0.05, we define it as a “true positive”. If the true protein abundance ratio is greater than 1, but the estimated ratio is not greater than 1 or the adjusted p-value is greater than 0.05, this protein is a “false negative”. All the adjusted p-values we used were corrected by the Benjamini-Hochberg method for each tool, which is the common correction method for all tools evaluated. In the large-scale mix data set, if the estimated protein meets the previous TP definition and the species belongs to Yeast, we define it as a “true positive”. If the protein does not satisfy the numerical condition but the species condition, it is a “false negative”. While the normalization and imputation methods employed by each tool are different, with this benchmark we aim to find what combination of parameters on each tool provides higher positive predicted values (PPV) and Negative predicted values (NPV). Since the toxicology data set is not a gold-standard data set, PPV and NPV cannot be calculated.

**Table 2:** PPVs and NPVs for Different Combinations of Parameters and Methods (Imputation and Normalization) on Protein Level^a^

Tool (imputation-normalization)	UPS spiked data set		Tool (imputation-normalization)	Large-scale data set (4:1 fold)		Large-scale data set (10:1 fold)	
	PPV	NPV		PPV	NPV	PPV	NPV
Proteus (MVL-NN)	0.97 (31 TP, total 2231)	0.998 (3 FN)	Proteus (MVL-NN)	0.62 (431 TP, total 3694)	0.972 (85 FN)	0.54 (475 TP, total 3403)	0.981 (48 FN)
prolfqua (GMI-RS)	0.91 (39 TP, total 2143)	0.999 (1 FN)	prolfqua (GMI-RS)	0.65 (544 TP, total 3043)	0.879 (267 FN)	0.54 (703 TP, total 3043)	0.938 (108 FN)
ProVision (ND-NN)	0.93 (38 TP, total 1987)	1.0 (1 FN)	ProVision (ND-NN)	0.62 (239 TP, total 2310)	0.851 (287 FN)	0.53 (400 TP, total 2310)	0.919 (126 FN)
LFQ-Analyst (QRILC-NN)	0.93 (38 TP, total 1988)	1.0 (0 FN)	LFQ-Analyst (QRILC-NN)	0.70 (263 TP, total 2108)	0.836 (284 FN)	0.59 (454 TP, total 2108)	0.931 (93 FN)
Eatomics (ND-limma VSN)	0.93 (38 TP, total 1826)	1.0 (0 FN)	Eatomics (ND-limma VSN)	0.63 (43 TP, total 1122)	0.992 (8 FN)	0.33 (49 TP, total 1041)	0.998 (2 FN)
ProStaR (DQ-GQA)	0.84 (37 TP, total 2238)	1.0 (0 FN)	ProStaR (DQ-GQA)	0.55 (304 TP, total 5521)	0.739 (1295 FN)	0.57 (504 TP, total 5521)	0.764 (1095 FN)
Perseus (GD-NN)	0.95 (38 TP, total 1946)	1.0 (0 FN)	Perseus (zero-NN)	0.65 (159 TP, total 1593)	0.950 (67 FN)	0.63 (314 TP, total 1629)	0.955 (51 FN)

a For all these tests, the input protein expression tables were generated by MaxQuant to perform the differential expression analysis. Only the best combination for each tool is presented. Supplementary Table 4 contains all combinations’ results. The definition of the best combination is that the higher the PPV with the same amount of TP proteins. If there is a large difference in the amount of TP proteins, the greater the amount of TP proteins, the better the combination.

In the UPS spiked data set, Perseus, Eatomics, LFQ-Analyst, and Provision detected most of the true positive proteins (38 UPS proteins), but Proteus achieved the highest PPV but with the lowest TP.^31^ Note that when filtering for an FDR of 5%, we must expect PPV ≥ 0.95, then all the combinations PPV ≥ 0.95 can be considered as good combinations (e.g., all Perseus combinations for UPS spiked data set). ProStaR achieved the lowest 84% PPV even if it had 100% NPV. Remarkably, most of the tools and combinations of parameters achieved Negative predicted values (NPV) higher than 0.95. Supplementary Table 4 shows large differences among combinations of parameters for the same tool (e.g., Proteus) resulting in different protein lists. For example, if a normalization method is applied with Proteus, more true positive proteins are DE quantified (33 versus 31 UPS proteins), while the number of false positives increases.

In addition, we compared the adjusted p-values obtained by the six different tools with the best combination of parameters (Best PPV and NPV results, Table 2) by Bland-Altman plot (Supplementary Figure 1). We did not try ProStaR because its default output does not display the list of adjusted p-values. The Bland-Altman plot^49^ is a robust method to assess the agreement between two quantitative methods; allowing one to measure the agreement between methods by studying the mean difference and constructing limits of the agreement.^49^ A lower difference is observed among the three tools: Perseus, Eatomics, and LFQ-Analyst, which can imply the results are alternative between them. However, Proteus and prolfqua adjusted p-values are different compared with other tools. When we compared the estimated log-fold change (log2FC) and the expected log-fold change for each tool, all Pearson correlations were higher than 0.85 (Supplementary Figure 2), which means that the log2FC is consistent with the expected value within a tool. It is important to note for Perseus users that the direct alternatives to the tool (ProVision, Eatomics, and LFQ-Analyst) will produce values between 0.7 and 0.8 Pearson correlation compared to Perseus.

We draw the boxplot of estimated log2-fold changes produced by each tool for background proteins in the UPS spiked data set (Supplementary Figure 3). Perseus achieves less dispersive log2FC values and is centered on 0 (−0.05 median, −0.16 lower quartile, and 0.04 upper quartile). Proteus and prolfqua produce more dispersive values (Proteus: 0.09 median, −0.05 lower quartile, and 0.24 upper quartile) and the latter had worse log2FC (−0.13 median, −0.26 lower quartile, and 0.03 upper quartile), as did ProStaR (−0.18 median, −0.31 lower quartile, and −0.05 upper quartile).

For the large-scale mix data set, prolfqua detected most of the true positive proteins (544 and 703) in both the 4:1 fold data set and 10:1 fold data set, but Perseus achieved better PPV in the former and LFQ-Analyst achieved better PPV in the latter. In the former data set (4:1 fold), ProStaR achieved the lowest 55% PPV but Eatomics returned the lowest 43 TP proteins. In the latter data set (10:1 fold), Eatomics achieved the lowest 33% PPV even though it had a 99.8% NPV, and it returned the lowest 49 TP proteins. Similarly, different combinations of parameters for the same tool produce different results. For example, when enabling the KNN imputation, LFQ-Analyst achieved the highest PPV (64%) while it recognized true positive proteins (335) are the lowest (Supplementary Table 4).

### 4.2 Peptide-Based Analysis

In addition to exploring the performance of these packages in analyzing MaxQuant protein results, we also explored R-package alternatives to analyze peptide-intensity data. Five packages (MSstats, Proteus, prolfqua, ProStaR, msqrob2) support the data analysis from peptide-level intensity tables (Table 1). We explored MSstats, Proteus, and msqrob2 with peptide-intensity results from quantms and MaxQuant. Previous benchmarks have been performed only using MaxQuant, making it difficult to carry the same conclusions to other quantification tools. quantms is a new cloud-based, open-source workflow that enables large-scale proteomics data analysis and generates peptide-level quantification and could benefit from this evaluation. The protein quantities have been generated by each R-package or tool starting from the peptide intensity table from quantms, and the uploaded tables by each tool can be viewed in Supplementary Table 2.

MSstats summarizes the peptide intensities into protein intensities, and linear modeling or Tukey’s median polish is then performed at the protein level.^20^ Similarly, the Proteus approach computes the protein-level intensities by calculating the mean of the three most intense peptides. Then, limma, which offers robust treatment of missing data, is used to perform the differential expression analysis. Furthermore, msqrob2 aggregates peptide intensities to protein expression values by the robust summarization method in the QFeatures package.^50^ We did not evaluate prolfqua and ProStaR because they lack the support of the mzTab or MSstats file format. Table 3 shows the benchmark of all combinations of parameters, quantification tool (MaxQuant, quantms), and DE package.

**Table 3:** PPVs and NPVs for Different Combinations of Parameters and Methods (Imputation and Normalization) on the Peptide Level^a^

Source	Tool (imputation-normalization)	UPS spiked data set		Tool (imputation-normalization)	Large-scale data set (4:1 fold)		Tool (imputation-normalization)	Large-scale data set (10:1 fold)	
		PPV	NPV		PPV	NPV		PPV	NPV
quantms	MSstats (NaN-Q)	0.91 (30 TP, total 1896)	0.995 (10 FN)	MSstats (NaN-NN)	0.86 (554 TP, total 2740)	0.934 (137 FN)	MSstats (NaN-NN)	0.73 (628 TP, total 2740)	0.966 (63 FN)
	Proteus (MVL-EM)	0.91 (33 TP, total 1905)	0.997 (6 FN)	Proteus (MVL-NN)	0.75 (386 TP, total 2587)	0.928 (147 FN)	Proteus (MVL-NN)	0.66 (450 TP, total 2587)	0.952 (88 FN)
	msqrob2 (NaN-CM)	0.80 (12 TP, total 1657)	0.990 (16 FN)	msqrob2 (NaN-NN)	0.87 (388 TP, total 2310)	0.953 (88 FN)	msqrob2 (NaN-NN)	0.82 (423 TP, total 2310)	0.970 (53 FN)
MaxQuant	MSstats (NaN-Q)	0.90 (36 TP, total 2116)	0.999 (1 FN)	MSstats (NaN-NN)	0.90 (589 TP, total 2576)	0.973 (47 FN)	MSstats (NaN-NN)	0.85 (619 TP, total 2478)	0.990 (18 FN)
	Proteus (MVL-EM)	0.89 (34 TP, total 2115)	1.0 (0 FN)	Proteus (MVL-NN)	0.64 (308 TP, total 2418)	0.964 (70 FN)	Proteus (MVL-NN)	0.55 (343 TP, total 2314)	0.976 (40 FN)
	msqrob2 (NaN-CM)	0.87 (33 TP, total 2116)	0.999 (1 FN)	msqrob2 (NaN-Q)	0.81 (325 TP, total 2232)	0.982 (33 FN)	msqrob2 (NaN-Q)	0.53 (348 TP, total 2232)	0.994 (10 FN)

a *: No shared peptides, >2 unique peptides per protein. Only the best combination for each tool is presented. Supplementary Table 5 contains all combinations’ results.

MSstats outperform the other packages msqrob2 and Proteus in the large-scale and UPS spiked data sets when using both quantification tools MaxQuant and quantms (Table 3). The only exception is the combination of UPS spiked data set and quantms, where Proteus outperforms MSstats. quantms and Proteus combinations returned more true positive proteins and higher PPV values than MaxQuant combinations.

**Figure 1:**
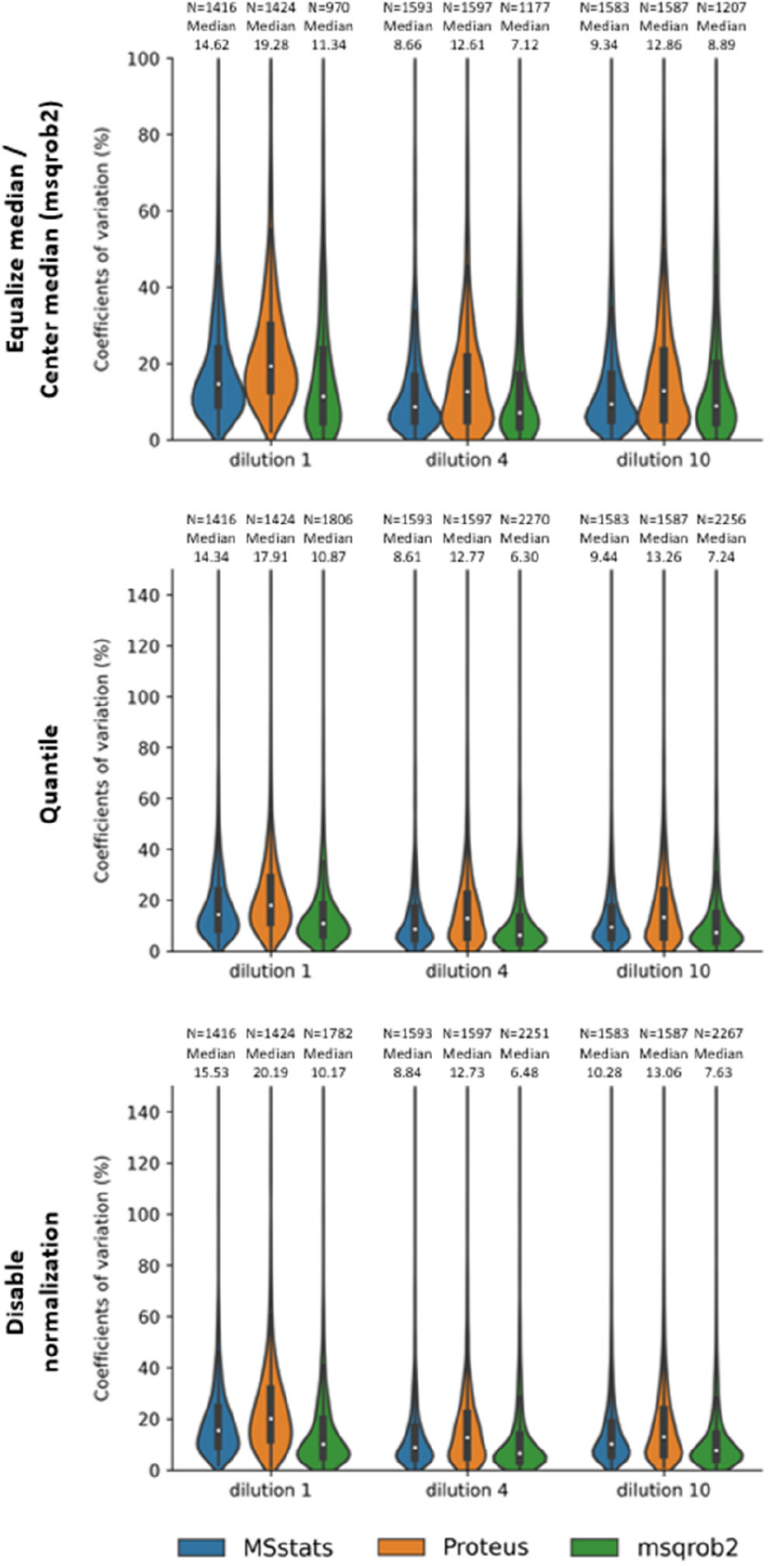
Coefficient of variation of three normalization methods based on the six replicates of the large-scale mix data set for MSstats, Proteus, and msqrob2. The MSstats and Proteus used default imputation methods NaN and MVL, respectively, and the msqrob2 did not use the imputation method. The underlying protein quantities have been generated by the tools starting from the intensity peptide table output of quantms.

We calculated the coefficient of variation (CV) distributions in Figure 1, which shows the CV distributions in at least 50% replicates for different tools and normalization methods. The CV is calculated from the standard deviation of protein intensities divided by the mean within a sample after normalization and aggregation by MSstats, Proteus, and msqrob2. Note that the intensity of the protein after aggregation generated by msqrob2 have negative values, and we deleted them when plotting. There are six technical replicates per sample in the large-scale mix data set, so we only calculate the CV of proteins quantified in at least 3 runs. From Figure 1, the MSstats package achieved a lower average CV of 15.0% across conditions compared with Proteus (average of 19.3%), and msqrob2 achieved the lowest average CV of 13.7%. Supplementary Figure 4 presents a box plot of the log2FC distribution produced by MSstats, msqrob2 and Proteus with different parameters combination. For low fold changes, msqrob2(NN) achieved a better-estimated log2FC (0.05 human proteins median and 2.02 Yeast proteins median) than others. It is worth noting that MSstats(NN) has smaller variances of log2FC values 1.30, and the values were reduced from MSstats (NN) 1.36 to MSstats (EM) 1.30 by median normalization. For high fold changes, msqrob2 (NN) also achieved a better-estimated log2FC with a median of −0.04 in Human and 3.26 in Yeast. On the contrary, the Proteus got a lower estimated log2FC with a median of 1.82 on low fold changes and 2.95 on high fold changes.

**Figure 2:**
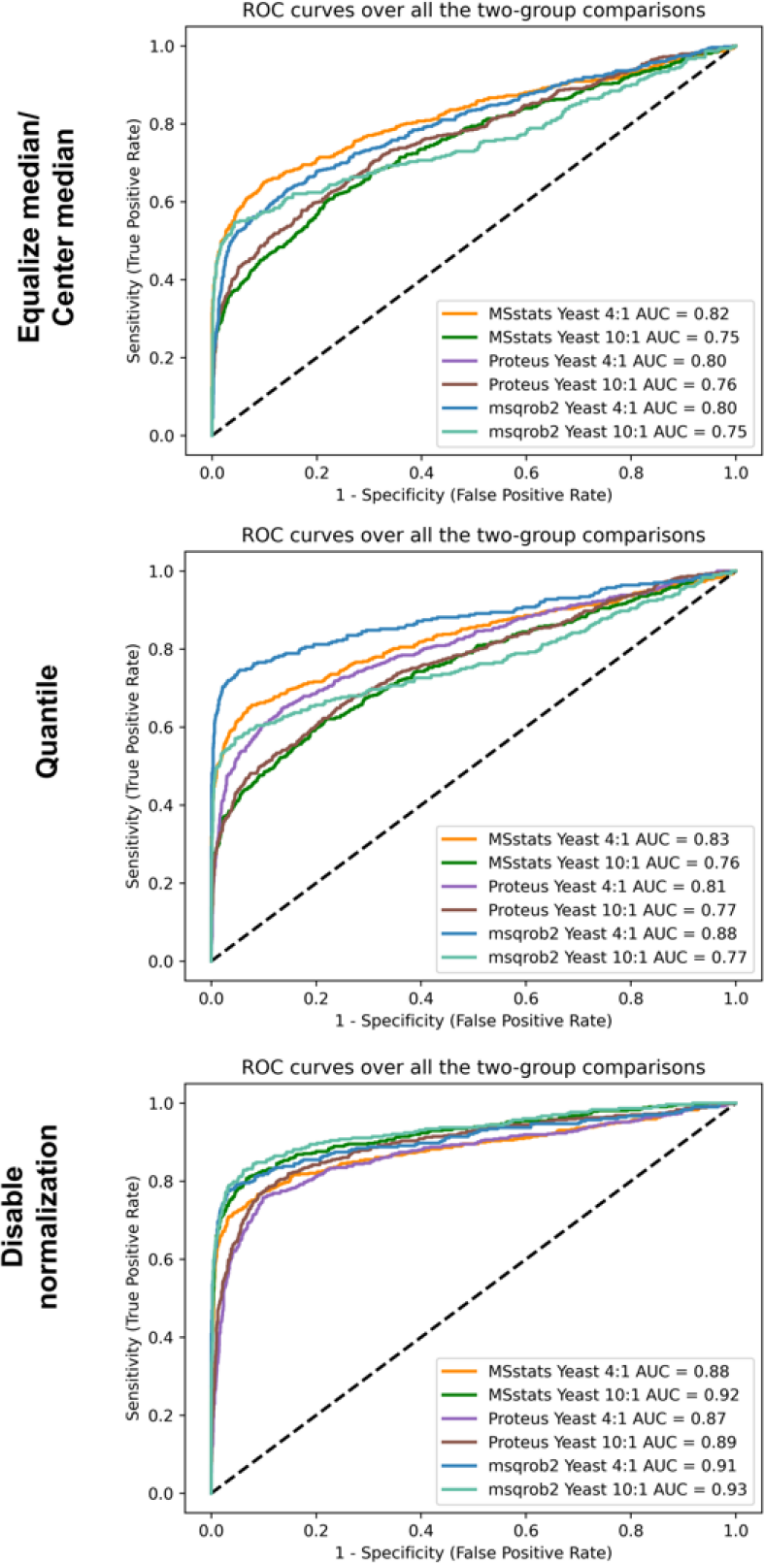
ROC plots from MSstats, msqrob2 and Proteus for large-scale mix data set analyzed with quantms. The figure highlights the sensitivity and specificity of the tools in classifying proteins with significant fold changes under different normalization settings. The underlying protein quantities have been generated starting from the peptide intensity table output of quantms.

For a large-scale mix data set, the true positive rates are straightforward, and we can plot ROC curves based on the adjusted p-values for each case to assess the performance of MSstats, msqrob2, and Proteus (Figure 2). We see that MSstats have higher accuracy, with area under the curve (AUC) values of 0.82 at a 4-fold change based on the median normalization method. However, msqrob2 performs better in other cases. Overall, MSstats and msqrob2 have similar performances for low fold change and they obtain the same number of differentially expressed proteins at a fixed FDR level when only the shared yeast proteins with adjusted p-value < 0.2 are considered (Supplementary Figure 5). For a 10-fold change, msqrob2 and MSstats yielded 257 TP, 71 FP proteins and 258 TP, 76 FP proteins, respectively, but Proteus only returns 252 TP proteins and 54 FP proteins at 1% FDR. For a 4-fold change, MSstats and msqrob2 achieve similar performances (MSstats 226 TP and 18 FP, msqrob2 230 TP and 20 FP) at 1% FDR. Proteus returns 221 TP and 18 FP proteins. The above differences should be within a reasonably acceptable range.

**Figure 3:**
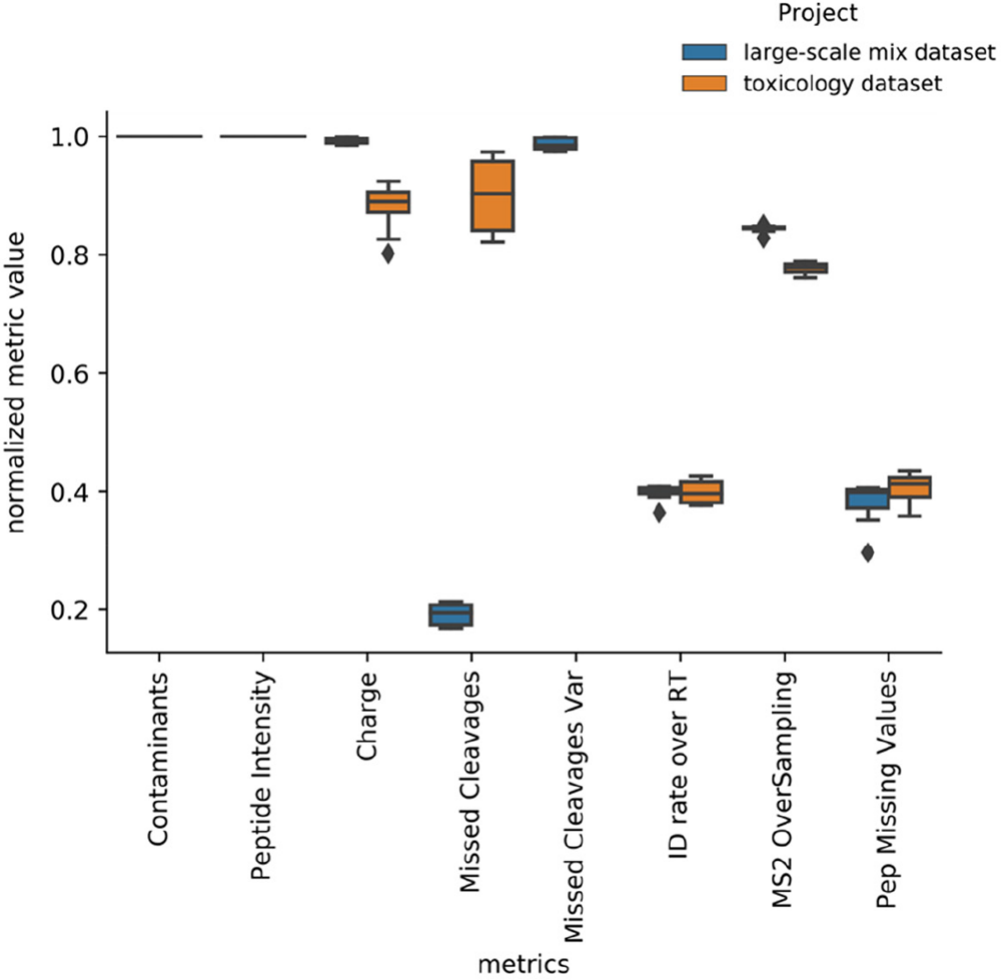
Distribution of metrics including contaminants, peptide intensity, charge, missed cleavages, identification rate over retention time, MS2 oversampling, and peptide missing values for the large-scale mix data set and toxicology data set analyzed with quantms. The normalized metric value (*y*-axis) is the normalized value for each specific metric between 0 and 1.

In addition to the previous two gold standard data sets, we analyzed the toxicology data set where no ratio is known between samples and no spike proteins have been used. Interestingly, in the original paper^33^ the authors did not use any package for normalization, missing value imputation, or DE analysis. Supplementary Figure 6 shows the CVs for all replicates of the toxicology data set. For all normalization methods, MSstats shows lower CV values than Proteus; and the values for both tools are quite high compared with the previous analysis large-scale mix data set (Proteus: 19.3%, MSstats: 15.0% in the large-scale mix data set; Proteus: 28.0%, MSstats: 16.7%, msqrob2: 13.7% in the toxicology data set). For msqrob2, the performance of this tool in the toxicology data set (average of 17.6%) is not as good as that in the large-scale mix data set (average of 13.7%). With a deeper look into the data sets, the large-scale mix data set, and the toxicology data set result from quantms we observed a significant variation in the number of missed cleavages and missing values (Figure 3). The quantms quality control heatmap reports of both data sets (Supplementary Figure 7A,B) show this variation at the level of the MS run file. This may be the reason why we observed major differences when analyzing the number of DE proteins at different FDR thresholds for different normalization methods (Supplementary Figure 8) and a little agreement among tools for the final DE proteins (Supplementary Figure 9). However, MSstats and msqrob2 returned the same number of differentially expressed proteins (147 DEP) at a 0.05 p-value threshold. Intriguingly, Proteus shared less than 20% in all normalization cases with the original author’s results while MSstats and the paper results shared around ∼40% of the reported proteins. While MSstats and msqrob2 perform relatively better for the toxicology data set, but the former performs better in the UPS spiked data set). We included these contradictory results to show that data quality may introduce more differences and variability in the results than the tool used to perform the DE analysis.

## 5 Conclusions

This study explores multiple R-packages and tools for differential expression data analysis of LFQ experiments based on protein and peptide intensity results. We explored eight different R or Shiny applications including MSstats, Proteus, prolfqua, ProVision, LFQ-Analyst, Eatomics, ProStaR, msqrob2, and the popular C# tool Perseus. Most of these packages (LFQ-Analyst, ProVision, Eatomics, prolfqua, msqrob2) have been created as an alternative to Perseus to analyze the output of MaxQuant in the R. MSstats, prolfqua, Proteus, msqrob2, and ProStaR can process not only protein intensity results but peptide-based results, making them more flexible and compatible with tools such as DIA-NN, quantms, Skyline, etc. MSstats is the most well-documented package and the one with more support. However, other packages like prolfqua have higher quality control capabilities, which are crucial in differential expression data analysis to detect variances and errors in specific samples or analytical steps.

The results highlighted that major differences for some tools and data sets are observed when protein level intensity from MaxQuant is used because the performance of some tools can fluctuate significantly depending on the analysis parameters and even the characteristics of the input data set. Most of the tools can correctly quantify true positives at low false positive rates, however, Perseus, prolfqua, and LFQ-Analyst were the tools that performed better among the benchmarked packages in the two data sets. We used Proteus, msqrob2, and MSstats to evaluate the performance of protein quantification based on peptide-level intensities data from the quantms workflow and MaxQuant. Overall, for both data sets analyzed (the large-scale mix data set and the toxicology data set) msqrob and MSstats provided lower CVs across replicates than Proteus and more accurate quantification of differential expression ratios in the large-scale mix data set. However, we observed that when the data sets presented higher CV values independently of the tools used for the analysis, the results were not consistent and difficult to compare across tools. This fact should trigger a more rigorous study including the possible development of guidelines to make the quantitative results reproducible and accurate, even if they pass the author’s specific statistical tests.

At present, there are many algorithms and tools for identifying and quantifying proteomic data and R-packages to perform differential expression analysis. However, there may be some tools and algorithms that have not been evaluated in this article for retrieval methods or other reasons. Each package and tool provide different methods for normalization, imputation, visualization, and quality control of the DE protein results. Due to the diversity of statistical methods, algorithms, and data sets, we do not evaluate all of them but select some of them for comparison. By summarizing and comparing existing tools, this work can serve as a starting point for comparisons of label-free quantitation.

## Supporting Information Available

LFQ-based peptide and protein intensity differential expression analysis, Supplementary Table 1: Normalization and imputation methods in the evaluated tools, Supplementary Table 2: The input information required to run each of the tools, Supplementary Table 3: The quality metrics in output files of tools, Supplementary Table 4: Positive predicted values (PPVs) and negative predicted values (NPVs) for different combinations of parameters and methods (imputation and normalization) on the protein level, Supplementary Table 5: PPVs and NPVs for different combinations of parameters and methods (imputation and normalization) on the peptide level, Supplementary Figure 1: Bland-Altman plots for adjusted p-value comparison between six tools, Supplementary Figure 2: Scatter plots of the true and estimated log fold changes produced by eight tools for the UPS spiked data set, Supplementary Figure 3: Box plots of estimated log2 fold changes for the background proteins, Supplementary Figure 4: Box plots of estimated log2 fold changes produced by MSstats, Proteus, and msqrob2 with different parameters combinations, Supplementary Figure 5: Comparison of DE analysis on the PXD007145 data set, Supplementary Figure 6: Coefficient of variation for MSstats, Proteus, and msqrob2 with different normalization methods for the peptide level analysis of PXD020248 with quantms, Supplementary Figure 7: Heatmap quality control reports for data sets PXD007145 and PXD020248, made by quantms, Supplementary Figure 8: The number of common DEP that returned at a certain p-value range for data set PXD020248 analyzed with quantms, Supplementary Figure 9: Overlap of DEP among MSstats, Proteus, and original results for data set PXD020248 analyzed with quantms (PDF)

## Abbreviations

AUC: area under the curve
CV: coefficient of variation
DE: differential expression
DEP: differentially expressed protein
EM: equalize medians
FDR: false discovery rate
GMI: group mean imputation
GQA: global quantile alignment
LFQ: label-free quantitation
MVL: mean-variance by limma
CM: center median
NN: no normalization
NPV: negative predictive values
PPV: positive predictive value
RS: robust scale
TMT: tandem mass tag
TP: true positive

## References

[ref1] Ankney J. A.; Muneer A.; Chen X. Relative and Absolute Quantitation in Mass Spectrometry-Based Proteomics. Annu. Rev. Anal Chem. (Palo Alto Calif) 2018, 11 (1), 49–77. 10.1146/annurev-anchem-061516-045357. 29894226

[ref2] Aebersold R.; Mann M. Mass-spectrometric exploration of proteome structure and function. Nature 2016, 537 (7620), 347–55. 10.1038/nature19949. 27629641

[ref3] Mann M.; Jensen O. N. Proteomic analysis of post-translational modifications. Nat. Biotechnol. 2003, 21 (3), 255–61. 10.1038/nbt0303-255. 12610572

[ref4] Gouw J. W.; Krijgsveld J.; Heck A. J. Quantitative proteomics by metabolic labeling of model organisms. Mol. Cell Proteomics 2010, 9 (1), 11–24. 10.1074/mcp.R900001-MCP200. 19955089 PMC2808257

[ref5] Bantscheff M.; Lemeer S.; Savitski M. M.; Kuster B. Quantitative mass spectrometry in proteomics: critical review update from 2007 to the present. Anal Bioanal Chem. 2012, 404 (4), 939–65. 10.1007/s00216-012-6203-4. 22772140

[ref6] Zhu W.; Smith J. W.; Huang C. M. Mass spectrometry-based label-free quantitative proteomics. J. Biomed. Biotechnol. 2010, 2010, 840518 10.1155/2010/840518. 19911078 PMC2775274

[ref7] Wong J. W.; Cagney G. An overview of label-free quantitation methods in proteomics by mass spectrometry. Methods Mol. Biol. 2010, 604, 273–83. 10.1007/978-1-60761-444-9_18. 20013377

[ref8] Nahnsen S.; Bielow C.; Reinert K.; Kohlbacher O. Tools for label-free peptide quantification. Mol. Cell Proteomics 2013, 12 (3), 549–56. 10.1074/mcp.R112.025163. 23250051 PMC3591650

[ref9] Cox J.; Mann M. MaxQuant enables high peptide identification rates, individualized p.p.b.-range mass accuracies and proteome-wide protein quantification. Nat. Biotechnol. 2008, 26 (12), 1367–72. 10.1038/nbt.1511. 19029910

[ref10] Kim S.; Pevzner P. A. MS-GF+ makes progress towards a universal database search tool for proteomics. Nat. Commun. 2014, 5, 5277 10.1038/ncomms6277. 25358478 PMC5036525

[ref11] Vaudel M.; Burkhart J. M.; Zahedi R. P.; Oveland E.; Berven F. S.; Sickmann A.; Martens L.; Barsnes H. PeptideShaker enables reanalysis of MS-derived proteomics data sets. Nat. Biotechnol. 2015, 33 (1), 22–4. 10.1038/nbt.3109. 25574629

[ref12] Umer H. M.; Audain E.; Zhu Y.; Pfeuffer J.; Sachsenberg T.; Lehtio J.; Branca R.; Perez-Riverol Y., Generation of ENSEMBL-based proteogenomics databases boosts the identification of non-canonical peptides. Bioinformatics 2022.38 1470 10.1093/bioinformatics/btab838 34904638 PMC8825679

[ref13] Fan J.; Saha S.; Barker G.; Heesom K. J.; Ghali F.; Jones A. R.; Matthews D. A.; Bessant C. Galaxy Integrated Omics: Web-based Standards-Compliant Workflows for Proteomics Informed by Transcriptomics. Mol. Cell Proteomics 2015, 14 (11), 3087–93. 10.1074/mcp.O115.048777. 26269333 PMC4638048

[ref14] Mehta S.; Easterly C. W.; Sajulga R.; Millikin R. J.; Argentini A.; Eguinoa I.; Martens L.; Shortreed M. R.; Smith L. M.; McGowan T.; Kumar P.; Johnson J. E.; Griffin T. J.; Jagtap P. D. Precursor Intensity-Based Label-Free Quantification Software Tools for Proteomic and Multi-Omic Analysis within the Galaxy Platform. Proteomes 2020, 8 (3), 15 10.3390/proteomes8030015. 32650610 PMC7563855

[ref15] Lin M. H.; Wu P. S.; Wong T. H.; Lin I. Y.; Lin J.; Cox J.; Yu S. H. Benchmarking differential expression, imputation and quantification methods for proteomics data. Brief Bioinform. 2022, 23 (3), bbac138 10.1093/bib/bbac138. 35397162

[ref16] Matzke M. M.; Brown J. N.; Gritsenko M. A.; Metz T. O.; Pounds J. G.; Rodland K. D.; Shukla A. K.; Smith R. D.; Waters K. M.; McDermott J. E.; Webb-Robertson B. J. A comparative analysis of computational approaches to relative protein quantification using peptide peak intensities in label-free LC-MS proteomics experiments. Proteomics 2013, 13 (3–4), 493–503. 10.1002/pmic.201200269. 23019139 PMC3775642

[ref17] Audain E.; Uszkoreit J.; Sachsenberg T.; Pfeuffer J.; Liang X.; Hermjakob H.; Sanchez A.; Eisenacher M.; Reinert K.; Tabb D. L.; Kohlbacher O.; Perez-Riverol Y. In-depth analysis of protein inference algorithms using multiple search engines and well-defined metrics. J. Proteomics 2017, 150, 170–182. 10.1016/j.jprot.2016.08.002. 27498275

[ref18] Al Shweiki M. R.; Monchgesang S.; Majovsky P.; Thieme D.; Trutschel D.; Hoehenwarter W. Assessment of Label-Free Quantification in Discovery Proteomics and Impact of Technological Factors and Natural Variability of Protein Abundance. J. Proteome Res. 2017, 16 (4), 1410–1424. 10.1021/acs.jproteome.6b00645. 28217993

[ref19] Ramus C.; Hovasse A.; Marcellin M.; Hesse A. M.; Mouton-Barbosa E.; Bouyssie D.; Vaca S.; Carapito C.; Chaoui K.; Bruley C.; Garin J.; Cianferani S.; Ferro M.; Van Dorssaeler A.; Burlet-Schiltz O.; Schaeffer C.; Coute Y.; Gonzalez de Peredo A. Benchmarking quantitative label-free LC-MS data processing workflows using a complex spiked proteomic standard dataset. J. Proteomics 2016, 132, 51–62. 10.1016/j.jprot.2015.11.011. 26585461

[ref20] Choi M.; Chang C. Y.; Clough T.; Broudy D.; Killeen T.; MacLean B.; Vitek O. MSstats: an R package for statistical analysis of quantitative mass spectrometry-based proteomic experiments. Bioinformatics 2014, 30 (17), 2524–6. 10.1093/bioinformatics/btu305. 24794931

[ref21] Tyanova S.; Temu T.; Sinitcyn P.; Carlson A.; Hein M. Y.; Geiger T.; Mann M.; Cox J. The Perseus computational platform for comprehensive analysis of (prote)omics data. Nat. Methods 2016, 13 (9), 731–40. 10.1038/nmeth.3901. 27348712

[ref22] Gierlinski M.; Gastaldello F.; Cole C.; Barton G. J. Proteus: an R package for downstream analysis of MaxQuant output. bioRxiv 2018, 416511 10.1101/416511.

[ref23] Wolski W. E.; Nanni P.; Grossmann J.; d’Errico M.; Schlapbach R.; Panse C. prolfqua: A Comprehensive R-package for Proteomics Differential Expression Analysis. J. Proteome Res. 2023, 22 (4), 1092–1104. 10.1021/acs.jproteome.2c00441. 36939687 PMC10088014

[ref24] Gallant J. L.; Heunis T.; Sampson S. L.; Bitter W. ProVision: a web-based platform for rapid analysis of proteomics data processed by MaxQuant. Bioinformatics 2020, 36 (19), 4965–4967. 10.1093/bioinformatics/btaa620. 32638008 PMC7723325

[ref25] Shah A. D.; Goode R. J. A.; Huang C.; Powell D. R.; Schittenhelm R. B. LFQ-Analyst: An Easy-To-Use Interactive Web Platform To Analyze and Visualize Label-Free Proteomics Data Preprocessed with MaxQuant. J. Proteome Res. 2020, 19 (1), 204–211. 10.1021/acs.jproteome.9b00496. 31657565

[ref26] Kraus M.; Mathew Stephen M.; Schapranow M. P. Eatomics: Shiny Exploration of Quantitative Proteomics Data. J. Proteome Res. 2021, 20 (1), 1070–1078. 10.1021/acs.jproteome.0c00398. 32954734

[ref27] Wieczorek S.; Combes F.; Lazar C.; Giai Gianetto Q.; Gatto L.; Dorffer A.; Hesse A. M.; Coute Y.; Ferro M.; Bruley C.; Burger T. DAPAR & ProStaR: software to perform statistical analyses in quantitative discovery proteomics. Bioinformatics 2017, 33 (1), 135–136. 10.1093/bioinformatics/btw580. 27605098 PMC5408771

[ref28] Goeminne L. J. E.; Sticker A.; Martens L.; Gevaert K.; Clement L. MSqRob Takes the Missing Hurdle: Uniting Intensity- and Count-Based Proteomics. Anal. Chem. 2020, 92 (9), 6278–6287. 10.1021/acs.analchem.9b04375. 32227882

[ref29] Sticker A.; Goeminne L.; Martens L.; Clement L. Robust Summarization and Inference in Proteome-wide Label-free Quantification. Mol. Cell Proteomics 2020, 19 (7), 1209–1219. 10.1074/mcp.RA119.001624. 32321741 PMC7338080

[ref30] Goeminne L. J.; Gevaert K.; Clement L. Peptide-level Robust Ridge Regression Improves Estimation, Sensitivity, and Specificity in Data-dependent Quantitative Label-free Shotgun Proteomics. Mol. Cell Proteomics 2016, 15 (2), 657–68. 10.1074/mcp.M115.055897. 26566788 PMC4739679

[ref31] Cox J.; Hein M. Y.; Luber C. A.; Paron I.; Nagaraj N.; Mann M. Accurate proteome-wide label-free quantification by delayed normalization and maximal peptide ratio extraction, termed MaxLFQ. Mol. Cell Proteomics 2014, 13 (9), 2513–26. 10.1074/mcp.M113.031591. 24942700 PMC4159666

[ref32] Hogrebe A.; von Stechow L.; Bekker-Jensen D. B.; Weinert B. T.; Kelstrup C. D.; Olsen J. V. Benchmarking common quantification strategies for large-scale phosphoproteomics. Nat. Commun. 2018, 9 (1), 1045 10.1038/s41467-018-03309-6. 29535314 PMC5849679

[ref33] Wang Z.; Karkossa I.; Grosskopf H.; Rolle-Kampczyk U.; Hackermuller J.; von Bergen M.; Schubert K. Comparison of quantitation methods in proteomics to define relevant toxicological information on AhR activation of HepG2 cells by BaP. Toxicology 2021, 448, 152652 10.1016/j.tox.2020.152652. 33278487

[ref34] Gentleman R. C.; Carey V. J.; Bates D. M.; Bolstad B.; Dettling M.; Dudoit S.; Ellis B.; Gautier L.; Ge Y.; Gentry J.; Hornik K.; Hothorn T.; Huber W.; Iacus S.; Irizarry R.; Leisch F.; Li C.; Maechler M.; Rossini A. J.; Sawitzki G.; Smith C.; Smyth G.; Tierney L.; Yang J. Y.; Zhang J. Bioconductor: open software development for computational biology and bioinformatics. Genome Biol. 2004, 5 (10), R80 10.1186/gb-2004-5-10-r80. 15461798 PMC545600

[ref35] Gatto L.; Christoforou A. Using R and Bioconductor for proteomics data analysis. Biochim. Biophys. Acta 2014, 1844 (1), 42–51. 10.1016/j.bbapap.2013.04.032. 23692960

[ref36] Gatto L.; Breckels L. M.; Naake T.; Gibb S. Visualization of proteomics data using R and bioconductor. Proteomics 2015, 15 (8), 1375–89. 10.1002/pmic.201400392. 25690415 PMC4510819

[ref37] Pascovici D.; Handler D. C.; Wu J. X.; Haynes P. A. Multiple testing corrections in quantitative proteomics: A useful but blunt tool. Proteomics 2016, 16 (18), 2448–53. 10.1002/pmic.201600044. 27461997

[ref38] MacLean B.; Tomazela D. M.; Shulman N.; Chambers M.; Finney G. L.; Frewen B.; Kern R.; Tabb D. L.; Liebler D. C.; MacCoss M. J. Skyline: an open source document editor for creating and analyzing targeted proteomics experiments. Bioinformatics 2010, 26 (7), 966–8. 10.1093/bioinformatics/btq054. 20147306 PMC2844992

[ref39] Ritchie M. E.; Phipson B.; Wu D.; Hu Y.; Law C. W.; Shi W.; Smyth G. K. limma powers differential expression analyses for RNA-sequencing and microarray studies. Nucleic Acids Res. 2015, 43 (7), e47 10.1093/nar/gkv007. 25605792 PMC4402510

[ref40] Huber W.; von Heydebreck A.; Sultmann H.; Poustka A.; Vingron M. Variance stabilization applied to microarray data calibration and to the quantification of differential expression. Bioinformatics 2002, 18 (Suppl 1), S96–S104. 10.1093/bioinformatics/18.suppl_1.S96. 12169536

[ref41] Demichev V.; Messner C. B.; Vernardis S. I.; Lilley K. S.; Ralser M. DIA-NN: neural networks and interference correction enable deep proteome coverage in high throughput. Nat. Methods 2020, 17 (1), 41–44. 10.1038/s41592-019-0638-x. 31768060 PMC6949130

[ref42] Bouyssie D.; Hesse A. M.; Mouton-Barbosa E.; Rompais M.; Macron C.; Carapito C.; Gonzalez de Peredo A.; Coute Y.; Dupierris V.; Burel A.; Menetrey J. P.; Kalaitzakis A.; Poisat J.; Romdhani A.; Burlet-Schiltz O.; Cianferani S.; Garin J.; Bruley C. Proline: an efficient and user-friendly software suite for large-scale proteomics. Bioinformatics 2020, 36 (10), 3148–3155. 10.1093/bioinformatics/btaa118. 32096818 PMC7214047

[ref43] Gatto L.; Gibb S.; Rainer J. MSnbase, Efficient and Elegant R-Based Processing and Visualization of Raw Mass Spectrometry Data. J. Proteome Res. 2021, 20 (1), 1063–1069. 10.1021/acs.jproteome.0c00313. 32902283

[ref44] Argentini A.; Goeminne L. J.; Verheggen K.; Hulstaert N.; Staes A.; Clement L.; Martens L. moFF: a robust and automated approach to extract peptide ion intensities. Nat. Methods 2016, 13 (12), 964–966. 10.1038/nmeth.4075. 27898063

[ref45] Hoffmann N.; Rein J.; Sachsenberg T.; Hartler J.; Haug K.; Mayer G.; Alka O.; Dayalan S.; Pearce J. T. M.; Rocca-Serra P.; Qi D.; Eisenacher M.; Perez-Riverol Y.; Vizcaino J. A.; Salek R. M.; Neumann S.; Jones A. R. mzTab-M: A Data Standard for Sharing Quantitative Results in Mass Spectrometry Metabolomics. Anal. Chem. 2019, 91 (5), 3302–3310. 10.1021/acs.analchem.8b04310. 30688441 PMC6660005

[ref46] Griss J.; Jones A. R.; Sachsenberg T.; Walzer M.; Gatto L.; Hartler J.; Thallinger G. G.; Salek R. M.; Steinbeck C.; Neuhauser N.; Cox J.; Neumann S.; Fan J.; Reisinger F.; Xu Q. W.; Del Toro N.; Perez-Riverol Y.; Ghali F.; Bandeira N.; Xenarios I.; Kohlbacher O.; Vizcaino J. A.; Hermjakob H. The mzTab data exchange format: communicating mass-spectrometry-based proteomics and metabolomics experimental results to a wider audience. Mol. Cell Proteomics 2014, 13 (10), 2765–75. 10.1074/mcp.O113.036681. 24980485 PMC4189001

[ref47] Pfeuffer J.; Sachsenberg T.; Alka O.; Walzer M.; Fillbrunn A.; Nilse L.; Schilling O.; Reinert K.; Kohlbacher O. OpenMS - A platform for reproducible analysis of mass spectrometry data. J. Biotechnol. 2017, 261, 142–148. 10.1016/j.jbiotec.2017.05.016. 28559010

[ref48] Eng J. K.; Jahan T. A.; Hoopmann M. R. Comet: an open-source MS/MS sequence database search tool. Proteomics 2013, 13 (1), 22–4. 10.1002/pmic.201200439. 23148064

[ref49] Giavarina D. Understanding Bland Altman analysis. Biochem Med. (Zagreb) 2015, 25 (2), 141–51. 10.11613/BM.2015.015. 26110027 PMC4470095

[ref50] Gatto L.; Vanderaa C. QFeatures: Quantitative features for mass spectrometry data. R package version 1.4.0; UCLouvain, 2021.

