# Supplementary material for: LFQ-Based Peptide and Protein Intensity Differential Expression Analysis
Source: J Proteome Res. 2023 May 23;22(6):2114–23. doi: 10.1021/acs.jproteome.2c00812 (PMC10243145; doi:10.1021/acs.jproteome.2c00812)
Supplement: Supplementary file 1 — pr2c00812_si_001.pdf [file pr2c00812_si_001.pdf]

Supplementary Notes

LFQ-based peptide and protein intensity differential expression analysis

Mingze Bai [1, 2, *], Jingwen Deng [1], Chengxin Dai [1,2], Julianus Pfeuffer [3,4], Timo Sachsenberg [5], Yasset Perez-Riverol [6, *]

[1] Chongqing Key Laboratory of Big Data for Bio Intelligence, Chongqing University of Posts and Telecommunications, Chongqing, China.

[2] State Key Laboratory of Proteomics, Beijing Proteome Research Center, National Center for Protein Sciences (Beijing), Beijing Institute of Life Omics, Beijing 102206, China.

[3] Algorithmic Bioinformatics, Freie Universität Berlin, Berlin 14195, Germany

[4] Visualization and Data Analysis, Zuse Institute Berlin, Berlin 14195, Germany

[5] Institute for Bioinformatics and Medical Informatics, University of Tübingen, Tübingen 72076, Germany

[6] European Molecular Biology Laboratory, European Bioinformatics Institute (EMBL-EBI), Wellcome Trust Genome Campus, Hinxton, Cambridge CB10 1SD, UK.

Corresponding authors: Yasset Perez-Riverol (yperez@ebi.ac.uk), Mingze Bai (baimz@cqupt.edu.cn)

Table of Contents

LFQ-based peptide and protein intensity differential expression analysis 1

Supplementary Table 1: Normalization and imputation methods in the evaluated tools. 3

Supplementary Table 2: The input information required to run each of the tools. 7

Supplementary Table 3: The quality metrics in output files of tools. 9

Supplementary Table 4: Positive predicted values (PPVs) and Negative predicted values (NPVs) for different combinations of parameters and methods (Imputation and Normalization) on the protein level. For all these tests, the input protein expression tables were generated by MaxQuant to perform the differential expression analysis. 10

Supplementary Table 5: Positive predicted values (PPVs) and Negative predicted values (NPVs) for different combinations of parameters and methods (Imputation and Normalization) on the peptide level. For all these tests, the input protein expression tables were generated by MaxQuant and quantms to perform the differential expression analysis. 13

Supplementary Figure 1: Bland-Altman plots for adjusted p-value comparison between six tools. In each plot, the x-axis represents the average adjusted p-values, and the y-axis represents the value difference of each protein (points in the plot) between tool pairs, the two red dotted lines represent 95% confidence interval limits. Plots in diagonal represent the adjusted p-values distribution of each tool. 15

Supplementary Figure 2: Scatter plots of the true and estimated log fold changes produced by eight tools for the UPS spiked dataset. (A) Proteus, (B) prolfqua, (C) ProVision, (D) LFQ-Analyst, (E) Eatomics, (F) ProStaR, (G) and Perseus. Only spiked proteins are plotted, and the Pearson correlation is shown. 16

Supplementary Figure 3: Box plots of estimated log2 fold changes for the background proteins.17

Supplementary Figure 4: Box plots of estimated log2 fold changes produced by MSstats, Proteus and msqrob2 with different parameters combinations for low fold changes (left panel) and high fold changes (right panel). 18

Supplementary Figure 5: Comparison of DE analysis on the PXD007145 dataset. The plot shows the number of differentially expressed proteins (DEP) that are returned by each tool at a certain FDR level, in which only the shared yeast proteins were assessed. The two vertical black lines indicate the 1 and 5% FDR levels. 19

Supplementary Figure 6: Coefficient of variation for MSstats, Proteus and msqrob2 with different normalization methods for the peptide level analysis of PXD020248 with quantms. 20

Supplementary Figure 7: Heatmap quality control reports for datasets PXD007145, and PXD020248, made by quantms. 21

Supplementary Figure 8: The number of common DEP that returned at a certain p-value range for dataset PXD020248 analyzed with quantms. 22

Supplementary Figure 9: Overlap of DEP between MSstats, Proteus and original results for dataset PXD020248 analyzed with quantms. 23

Supplementary Table 1: Normalization and imputation methods in the evaluated tools.

	Normalization methods	Imputation methods
MSstats	• Equalize median (default): represents constant normalization (equalizing the medians) based on reference signals is performed.<br>• Quantile: represents quantile normalization based on reference signals.<br>• Global standards: represents normalization with global standards proteins.	• Accelerated failure time model (default): censored missing values will be imputed by the Accelerated Failure Time model.<br>• cutoffCensored: cutoff value for AFT model.<br>• censoredInt: missing values are censored or at random.<br>• MaxQuantileforCensored: missing values are censored or at random.
msqrob2	• quantiles: applies quantile normalisation, as implemented in normalize.quantiles of the preprocessCore package.<br>• quantiles.robust: applies robust quantile normalization, as implemented in normalize.quantiles.robust of the preprocessCore package.<br>• vsn: uses the vsn2 function from the vsn package.<br>• center.median: translates the respective sample (column) intensities according to the column median from MSnbase package.<br>• center.mean: translates the respective sample (column) intensities according to the column mean from MSnbase package.<br>• max: each feature's reporter intensity is divided by the maximum from MSnbase package.<br>• sum: each feature's reporter intensity is divided by the sum from MSnbase package.<br>• div.mean: divide by the column means.<br>• div.median: divide by the column medians.<br>• diff.median: centers all samples (columns) so that they all match the grand median by subtracting the respective columns medians differences to the grand median.	X
ProStaR	• Global Quantile alignment: the Quantile of the intensity distributions of all the samples are equated, as proposed by the	• None(default)<br>• Det quantile (recommended): it proposes to impute each missing value within a given

	normalize.quantiles() of preprocessCore.<br>• Quantile Centering: a given quantile of the intensity distribution. It proposes to shift the sample distributions (either all of them at once, or within each condition at a time) to align a specific quantile.<br>• Mean Center: sample intensity distributions are aligned on their mean intensity values (and optionally, the variance distributions are equated to one).<br>• Column sums: the total intensity values of all the samples are equated. The rationale behind is to normalize according to the total amount of biological material within each sample.<br>• vsn: (variance stabilizing normalization) from vsn package. Its objective is to have the analyte variance independent of the intensity.<br>• LOESS: The intensity values are normalized by means of a local regression model of the difference of intensities as function of the mean intensity value, as implemented in the normalizeCyclicLoess() from limma package.	sample by a deterministic value (usually a low value).<br>• slsa (structured least square adaptative, recommended): it is a regression-based imputation method which account for a possible hierarchical design. It does not require any parameter tuning.<br>• KNN: (K-Nearest Neighbors) proposes to estimate each missing value by the mean of the observed values of other proteins with a similar intensity pattern (called neighbors). One only must tune K, the number of neighbors to account for. Fixed value: as an alternative, it is possible for the user to tune the imputation with a specific value that will be used in all samples of all conditions. Although appealing for its simplicity, this method may lead to important data distortion, so it is advised to use detQuantile algorithm whenever possible.
Proteus	• Equalize median (default): the function normalizeData() can normalize peptide or protein data. After this step, median sample intensities will be equal.<br>• Quantile: it works with normalizeQuantiles from limma package.	• Mean-variance (by limma package): limma offers an advantage over random imputation methods by borrowing information across peptides or proteins and using the mean-variance relationship to estimate variance where data are missing.
prolfqua	• robust scale: it uses a robust version of the z-score.	• Group mean imputation: it computes contrasts with group mean imputation (directly from data).
ProVision	X	• Normal distribution like Perseus: imputation method normal distributions as Perseus (0.3 width, 1.8 downshift).
LFQ-Analyst	X	• Perseus-type (default): imputation method normal distributions as Perseus (0.3 width, 1.8 downshift).<br>• KNN: missing values replace by nearest neighbor averaging technique.

		• bpca: Bayesian missing value imputation.<br>• QRILC: a missing data imputation method that performs the imputation of left-censored missing data using random draws from a truncated distribution with parameters estimated using quantile regression.<br>• MLE: This function performs missing values imputation using the EM algorithm from imputeLCMD package.<br>• MinDet: this function performs missing values imputation by the minimum value observed from imputeLCMD package.<br>• MinProb: performs the imputation of left-censored missing data by random draws from a Gaussian distribution centered to a minimal value. Considering an expression data matrix with n samples and p features, for each sample, the mean value of the Gaussian distribution is set to a minimal observed value in that sample.<br>• min: replaces the missing values by the smallest non-missing value in the data.<br>• zero: replaces the missing values by 0.
Eatomics	• VSN: in the case of iBAQ values, Eatomics performs automatic normalization via limma's normalizeVSN() function.	• perseus-like (default): imputation method normal distributions as Perseus (0.3 width, 1.8 downshift)<br>• KNN: k-nearest-neighbour from the impute package.<br>• MinDet: this function performs missing values imputation by the minimum value observed from imputeLCMD package.<br>• QRILC: this function performs missing values imputation-based quantile regression from imputeLCMD package.
Perseus	• Multiple algorithms (Z-score, Remove batch effect, Un-Z-score)	• A down-shifted Gaussian distribution (recommended): a custom implementation of Perseus' sampling from a down-shifted Gaussian distribution (implemented by Matthias Ziehm) with default parameters of width = 0.3 and shift = 1.8.<br>• constant: this function performs missing values imputation by customized constant

		value.<br>• ImputeLCMD: from imputeLCMD package.

Supplementary Table 2: The input information required to run each of the tools.

Tool	Minimum information required<br>file name (required columns)	Additional information<br>file name(columns)
MSstats	*[quantms]* out_msstats.csv<br>(ProteinName, Intensity, PeptideSequence, PrecursorCharge, IsotopeLabelType, Condition, BioReplicate, Run, Fraction)<br><br>*[MaxQuant]* proteinGroups.txt + evidence.txt + annotation.csv<br>(all columns) + (Sequence, Modified sequence, Proteins, Intensity, Raw file, Experiment, Charge, Protein group IDs) + (Raw.file, Condition, Experiment, TechReplicate)	*[quantms]* out_msstats.csv<br>FragmentIon, ProductCharge, Reference<br><br>*[MaxQuant]* proteinGroups.txt + evidence.txt + annotation.csv<br>(all columns) + (evidence.txt except basic information part) + (IsotopeLabelType, BioReplicate)
msqrob2	*[quantms]* .mzTab + annotation.csv<br>(accession, peptide_abundance_study_variable) + (run, treatment)<br><br>*[MaxQuant* peptides.txt + annotation.csv<br>(Proteins, Intensity) + (Name, Experiment, treatment)	*[quantms]* .mzTab + annotation.csv<br>(.mzTab except basic information part) + ()<br><br>*[MaxQuant]* peptides.txt + annotation.csv + proteinGroups.txt<br>(peptides.txt except basic information part) + (Fraction) + (Protein IDs / Majority protein IDs, Only identified by site)
ProStaR	proteinGroups.txt + manual annotation<br>(Protein IDs, Intensity / LFQ intensity) + (Condition)	proteinGroups.txt + manual annotation<br>proteinGroups.txt + manual annotation<br>(proteinGroups.txt except basic information part) + (Bio.Rep, Tech.Rep, Analyt.Rep)
Proteus	*[quantms]* out_proteus.csv + metadata.csv<br>(PeptideSequence, modified_sequence, protein, intensity, experiment) + (experiment, measure, sample, condition)<br><br>*[MaxQuant]* proteinGroups.txt + metadata.csv<br>(Majority protein IDs, Intensity, Only identified by site, Reverse, Potential contaminant) + (experiment, sample, condition)	*[quantms]* out_proteus.csv + metadata.csv<br>(modifications, protein_group, charge, sequence, accession) + (replicate)<br><br>*[MaxQuant]* proteinGroups.txt + metadata.csv<br>(proteinGroups.txt except basic information part) + (measure, replicate)

prolfqua	proteinGroups.txt + inputAnnotation.txt (Protein IDs, LFQ intensity, Intensity, Fasta headers, Peptides, MS/MS count, id) + (raw.file, condition, replicate)	proteinGroups.txt + inputAnnotation.txt (proteinGroups.txt except basic information part) + ()
ProVision	proteinGroups.txt + manual annotation (Majority protein IDs, Intensity / LFQ intensity, Unique peptides, Only identified by site, Reverse, Potential contaminant) + (ID, annotation, axisLabels)	proteinGroups.txt + manual annotation (proteinGroups.txt except basic information part) + ()
LFQ-Analyst	proteinGroups.txt + experimentalDesign.txt (Protein IDs, Intensity / LFQ intensity, Protein names, Gene names, Razor + unique peptides, Only identified by site, Reverse, Potential contaminant) + (label, condition, replicate)	proteinGroups.txt + experimentalDesign.txt (proteinGroups.txt except basic information part) + ()
Eatomics	proteinGroups.txt + ClinicalData.txt (Protein IDs, Majority protein IDs, LFQ intensity, Gene names, Only identified by site, Reverse, Potential contaminant) + (PatientID, condition)	proteinGroups.txt + ClinicalData.txt (proteinGroups.txt except basic information part) + (replicate)
Perseus	proteinGroups.txt + manual annotation (Protein IDs, Intensity / LFQ intensity) + (Categorical annotation rows)	proteinGroups.txt + manual annotation (proteinGroups.txt except basic information part)

Supplementary Table 3: The quality metrics in output files of tools.

Tool	*Quality metrics*
MSstats	Protein, Label, log2FC, SE, Tvalue, DF, pvalue, adj.pvalue, issue, MissingPercentage, ImputationPercentage
msqrob2	logFC, se, df, t, pval, adjPval
ProStaR	id, logFC (condition1_vs_condition2), P_Value (condition1_vs_condition2), isDifferential (condition1_vs_condition2), Protein_IDs
Proteus	protein, logFC, AveExpr, t, P.Value, adj.P.Val, B, significant, mean_condition1, mean_condition2, ngood_condition1, ngood_condition2
prolfqua	modelName, subject_Id, protein_Id, contrast, diff, std.error, avgAbd, statistic, df, p.value, conf.low, conf.high, sigma, FDR
ProVision	pValue, qValue, EffectSize, X.condition1. condition2., significant, GeneName, UniprotID
LFQ-Analyst	Gene Name, Protein IDs, condition1_vs_condition2 fold change, significant, imputed, num_Nas, Protein.names
Eatomics	Gene names, logFC, AveExpr, t, P.Value, adj.P.Val, B, Majority protein IDs, Protein IDs
Perseus	LFQ intensity, Only identified by site, Reverse, Potential contaminant, Student's T-test Significant condition1_condition2, Student's T-test significant, Peptides, Razor + unique peptides, Unique peptides, Sequence coverage [%], Unique + razor sequence coverage [%], Unique sequence coverage [%], Mol. weight [kDa], Q-value, Score, Intensity, MS/MS count, -Log Student's T-test p-value condition1_condition2, Student's T-test q-value condition1_condition2, Student's T-test Difference condition1_condition2, Student's T-test Test statistic condition1_condition2, Protein IDs, Majority protein IDs, id

Supplementary Table 4: Positive predicted values (PPVs) and Negative predicted values (NPVs) for different combinations of parameters and methods (Imputation and Normalization) on the protein level. For all these tests, the input protein expression tables were generated by MaxQuant to perform the differential expression analysis.

	**UPS spiked dataset**		**large-scale mix dataset (4:1 fold)**		**large-scale mix dataset (10:1 fold)**	
**Tool (Imputation – Normalization)**	**PPV**	**NPV**	**PPV**	**NPV**	**PPV**	**NPV**
**Proteus (MVL - EM)**	0.85 (33 TP, total 2231)	0.999 (1 FN)	0.60 (428 TP, total 3694)	0.970 (88 FN)	0.39 (467 TP, total 3403)	0.975 (56 FN)
**Proteus (MVL - Q)**	0.85 (33 TP, total 2231)	0.999 (1 FN)	0.61 (430 TP, total 3694)	0.971 (86 FN)	0.40 (468 TP, total 3403)	0.975 (55 FN)
**Proteus (MVL - NN)**	0.97 (31 TP, total 2231)	0.998 (3 FN)	0.62 (431 TP, total 3694)	0.972 (85 FN)	0.54 (475 TP, total 3403)	0.981 (48 FN)
**prolfqua (NN - RS)**	0.90 (35 TP, total 2136)	0.999 (1 FN)	0.68 (539 TP, total 2856)	0.916 (174 FN)	0.41 (356 TP, total 2415)	0.975 (39 FN)
**prolfqua (GMI – RS)**	0.91 (39 TP, total 2143)	0.999 (1 FN)	0.65 (544 TP, total 3043)	0.879 (267 FN)	0.54 (703 TP, total 3043)	0.938 (108 FN)
**ProVision (ND - NN)**	0.93 (38 TP, total 1987)	1.0 (0 FN)	0.62 (239 TP, total 2310)	0.851 (287 FN)	0.53 (400 TP, total 2310)	0.919 (126 FN)
**LFQ-Analyst (ND - NN)**	0.93 (38 TP, total 1988)	1.0 (0 FN)	0.63 (260 TP, total 2108)	0.831 (287 FN)	0.57 (453 TP, total 2108)	0.928 (94 FN)

**LFQ-Analyst (KNN - NN)**	0.92 (36 TP, total 1988)	0.998 (2 FN)	0.54 (128 TP, total 2108)	0.776 (419 FN)	0.64 (335 TP, total 2108)	0.866 (212 FN)
**LFQ-Analyst (zero – NN)**	0.94 (31 TP, total 1988)	0.996 (7 FN)	0.59 (260 TP, total 2108)	0.828 (287 FN)	0.58 (487 TP, total 2108)	0.953 (60 FN)
**LFQ-Analyst (QRILC – NN)**	0.93 (38 TP, total 1988)	1.0 (0 FN)	0.70 (263 TP, total 2108)	0.836 (284 FN)	0.59 (454 TP, total 2108)	0.931 (93 FN)
**Eatomics (ND- limma VSN)**	0.93 (38 TP, total 1826)	1.0 (0 FN)	0.63 (43 TP, total 1122)	0.992 (8 FN)	0.33 (49 TP, total 1041)	0.998 (2 FN)
**Eatomics (KNN – limma VSN)**	0.93 (38 TP, total 1826)	1.0 (0 FN)	0.63 (43 TP, total 1122)	0.992 (8 FN)	0.33 (49 TP, total 1041)	0.998 (2 FN)
**Eatomics (QRILC – limma VSN)**	0.93 (7 TP, total 1826)	1.0 (0 FN)	0.63 (43 TP, total 1122)	0.992 (8 FN)	0.33 (49 TP, total 1041)	0.998 (2 FN)
**ProStaR (DQ - GQA)**	0.84 (37 TP, total 2238)	1.0 (0 FN)	0.55 (304 TP, total 5521)	0.739 (1295 FN)	0.57 (504 TP, total 5521)	0.764 (1095 FN)
**ProStaR (DQ - NN)**	0.65 (37 TP, total 2248)	1.0 (0 FN)	0.36 (272 TP, total 5521)	0.722 (1327 FN)	0.51 (488 TP, total 5521)	0.757 (1111 FN)
**Perseus (NaN - NN)**	0.93 (27 TP, total 1936)	0.999 (1 FN)	0.77 (89 TP, total 1471)	0.975 (34 FN)	0.49 (96 TP, total 1335)	0.975 (29 FN)
**Perseus (GD – NN)**	0.95 (38 TP, total 1946)	1.0 (0 FN)	0.59 (107 TP, total 1483)	0.988 (16 FN)	0.23 (82 TP, total 1524)	0.995 (6 FN)
**Perseus**	0.94	0.996	0.65	0.950	0.63	0.955

**(zero - NN)**	(31 TP, total 1946)	(7 FN)	(159 TP, total 1593)	(67 FN)	(314 TP, total 1629)	(51 FN)

Supplementary Table 5: Positive predicted values (PPVs) and Negative predicted values (NPVs) for different combinations of parameters and methods (Imputation and Normalization) on the peptide level. For all these tests, the input protein expression tables were generated by MaxQuant and quantms to perform the differential expression analysis.

**Source**	**Tool** (Imputation – Normalization)	**UPS spiked dataset**		**Large-scale dataset (4:1 fold)**		**Large-scale dataset (10:1 fold)**	
		**PPV**	**NPV**	**PPV**	**NPV**	**PPV**	**NPV**
**quantms**	**MSstats (NaN-EM)**	0.90 (28 TP, total 1896)	0.994 (12 FN)	0.86 (537 TP, total 2740)	0.927 (154 FN)	0.62 (608 TP, total 2740)	0.952 (83 FN)
	**MSstats (NaN-Q)**	0.91 (30 TP, total 1896)	0.995 (10 FN)	0.85 (536 TP, total 2740)	0.926 (155 FN)	0.62 (609 TP, total 2740)	0.953 (82 FN)
	**MSstats (NaN-NN)**	0.80 (8 TP, total 1896)	0.983 (32 FN)	0.86 (554 TP, total 2740)	0.934 (137 FN)	0.73 (628 TP, total 2740)	0.966 (63 FN)
	**Proteus (MVL-EM)**	0.92 (33 TP, total 1905)	0.997 (6 FN)	0.75 (365 TP, total 2587)	0.920 (168 FN)	0.57 (432 TP, total 2535)	0.940 (106 FN)
	**Proteus (MVL-Q)**	0.92 (33 TP, total 1905)	0.997 (6 FN)	0.75 (362 TP, total 2587)	0.918 (171 FN)	0.57 (428 TP, total 2535)	0.938 (110 FN)
	**Proteus (MVL-NN)**	0.93 (28 TP, total 1905)	0.994 (11 FN)	0.75 (386 TP, total 2587)	0.928 (147 FN)	0.66 (450 TP, total 2587)	0.952 (88 FN)
	**msqrob2 (NaN-CM)**	0.80 (12 TP, total 1657)	0.990 (16 FN)	0.87 (381 TP, total 2310)	0.949 (95 FN)	0.70 (413 TP, total 2310)	0.963 (63 FN)
	**msqrob2 (NaN-Q)**	0.80 (12 TP, total 1656)	0.990 (16 FN)	0.88 (374 TP, total 2310)	0.946 (102 FN)	0.69 (413 TP, total 2310)	0.963 (63 FN)
	**msqrob2 (NaN-NN)**	1.0 (2 TP, total	0.984 (26 FN)	0.87 (388 TP,	0.953 (88 FN)	0.82 (423 TP, total	0.970 (53 FN)

		1655)		total 2310)		2310)	
**MaxQuant**	**MSstats**<br>**(NaN-EM)**	0.88<br>(36 TP, total 2116)	0.999<br>(1 FN)	0.89<br>(584 TP, total 2576)	0.973<br>(52 FN)	0.75<br>(618 TP, total 2478)	0.989<br>(19 FN)
	**MSstats**<br>**(NaN-Q)**	0.90<br>(36 TP, total 2116)	0.999<br>(1 FN)	0.89<br>(586 TP, total 2576)	0.974<br>(50 FN)	0.76<br>(619 TP, total 2478)	0.989<br>(18 FN)
	**MSstats**<br>**(NaN-NN)**	0.97<br>(30 TP, total 2116)	0.997<br>(7 FN)	0.90<br>(589 TP, total 2576)	0.976<br>(47 FN)	0.85<br>(619 TP, total 2478)	0.990<br>(18 FN)
	**Proteus**<br>**(MVL-EM)**	0.89<br>(34 TP, total 2115)	1.0<br>(0 FN)	0.62<br>(293 TP, total 2418)	0.956<br>(85 FN)	0.45<br>(334 TP, total 2314)	0.969<br>(49 FN)
	**Proteus**<br>**(MVL-Q)**	0.87<br>(33 TP, total 2115)	0.999<br>(1 FN)	0.63<br>(293 TP, total 2418)	0.956<br>(85 FN)	0.44<br>(335 TP, total 2314)	0.969<br>(48 FN)
	**Proteus**<br>**(MVL-NN)**	0.91<br>(32 TP, total 2115)	0.999<br>(2 FN)	0.64<br>(308 TP, total 2418)	0.964<br>(70 FN)	0.55<br>(343 TP, total 2314)	0.976<br>(40 FN)
	**msqrob2**<br>**(NaN-CM)**	0.87<br>(33 TP, total 2116)	0.999<br>(1 FN)	0.80<br>(324 TP, total 2233)	0.981<br>(34 FN)	0.50<br>(348 TP, total 2233)	0.994<br>(10 FN)
	**msqrob2**<br>**(NaN-Q)**	0.89<br>(32 TP, total 2115)	0.999<br>(1 FN)	0.81<br>(325 TP, total 2232)	0.982<br>(33 FN)	0.53<br>(348 TP, total 2232)	0.994<br>(10 FN)
	**msqrob2**<br>**(NaN-NN)**	0.89<br>(31 TP, total 2114)	0.999<br>(2 FN)	0.79<br>(321 TP, total 2233)	0.980<br>(37 FN)	0.52<br>(348 TP, total 2233)	0.994<br>(10 FN)

Supplementary Figure 1: Bland-Altman plots for adjusted p-value comparison between six tools. In each plot, the x-axis represents the average adjusted p-values, and the y-axis represents the value difference of each protein (points in the plot) between tool pairs, the two red dotted lines represent 95% confidence interval limits. Plots in diagonal represent the adjusted p-values distribution of each tool.

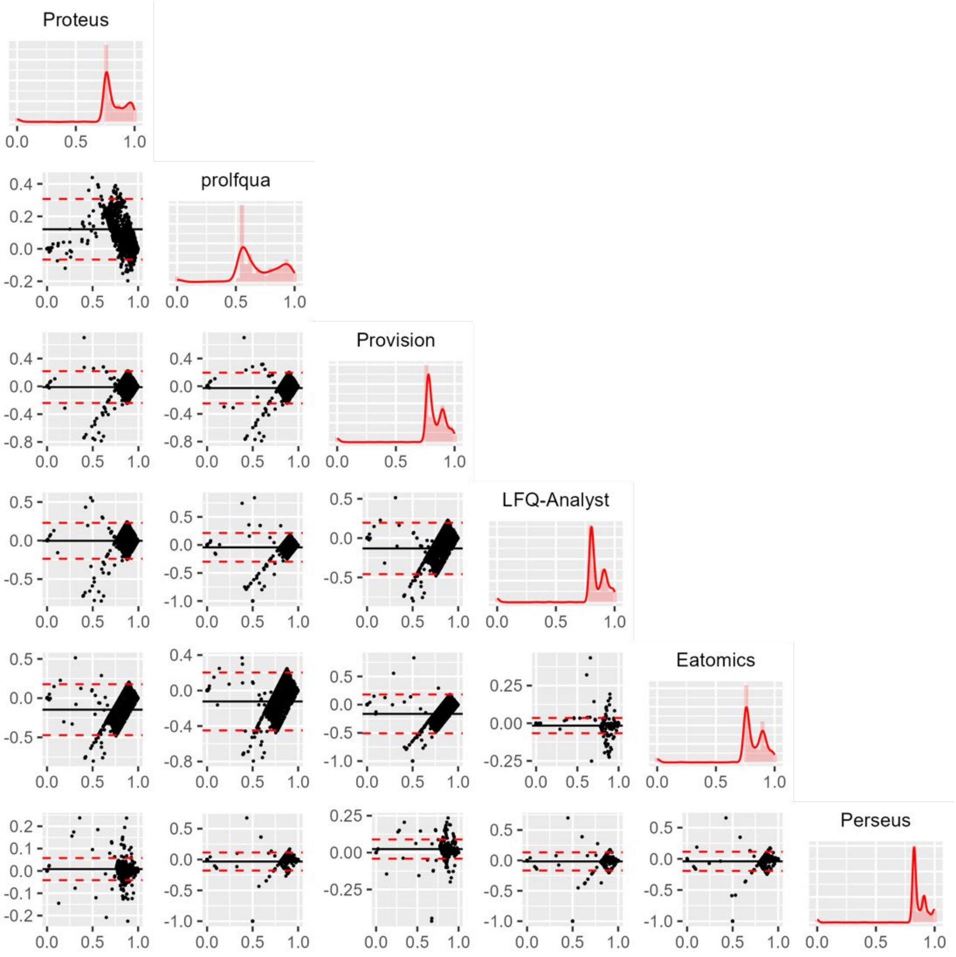

Supplementary Figure 2: Scatter plots of the true and estimated log fold changes produced by eight tools for the UPS spiked dataset. (A) Proteus, (B) prolfqua, (C) ProVision, (D) LFQ-Analyst, (E) Eatomics, (F) ProStaR, (G) and Perseus. Only spiked proteins are plotted, and the Pearson correlation is shown.

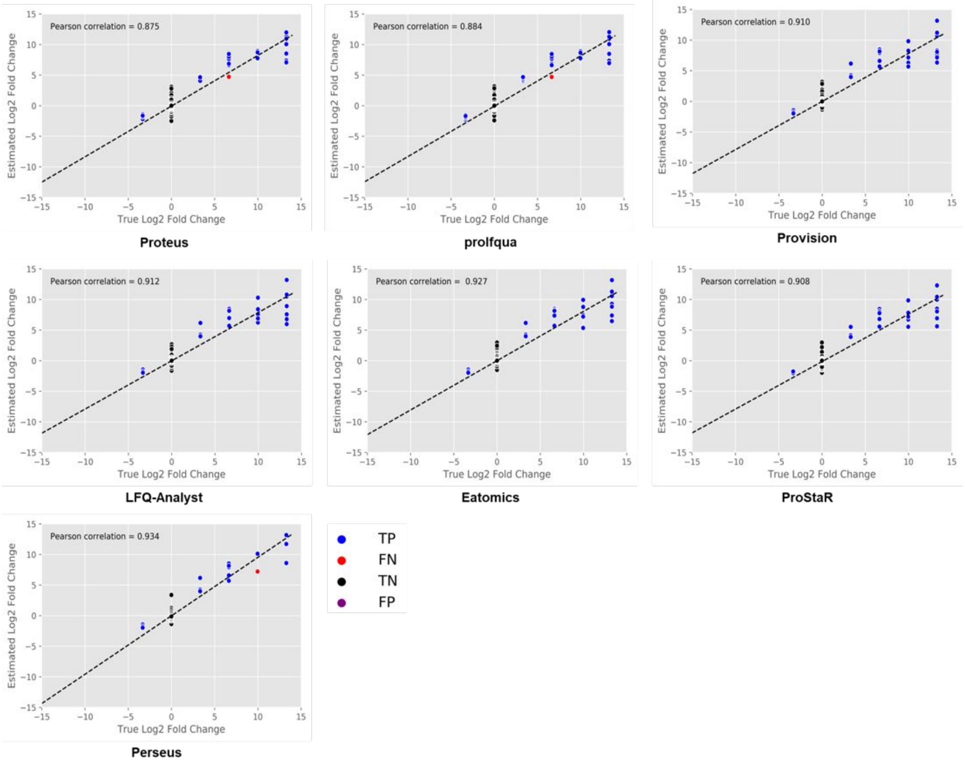

Supplementary Figure 3: Box plots of estimated log2 fold changes for the background proteins.

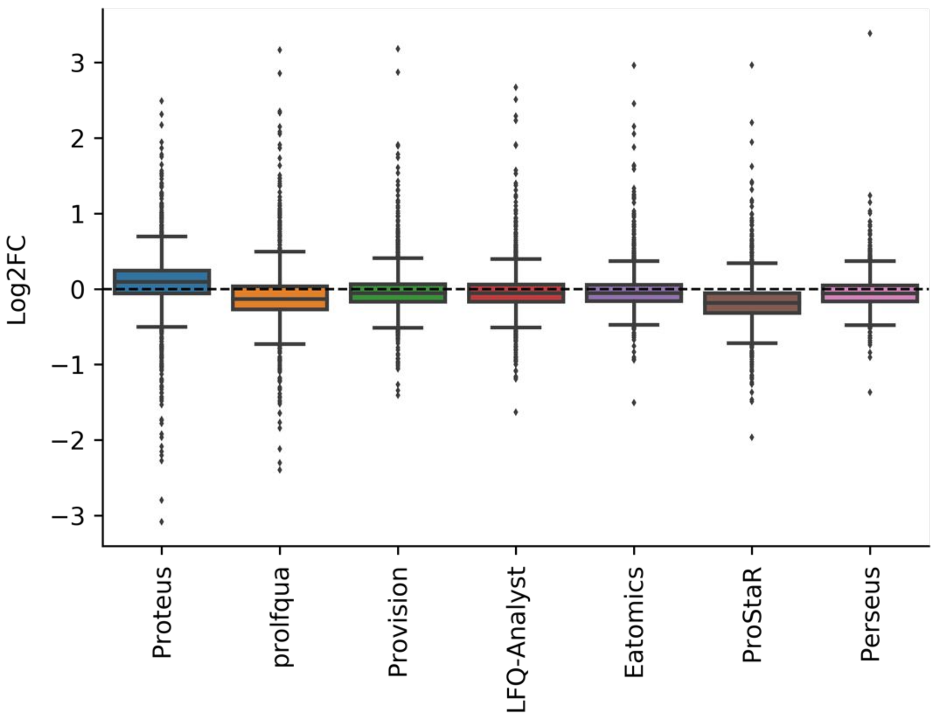

Supplementary Figure 4: Box plots of estimated log2 fold changes produced by MSstats, Proteus and msqrob2 with different parameter combinations for low fold changes (left panel) and high fold changes (right panel).

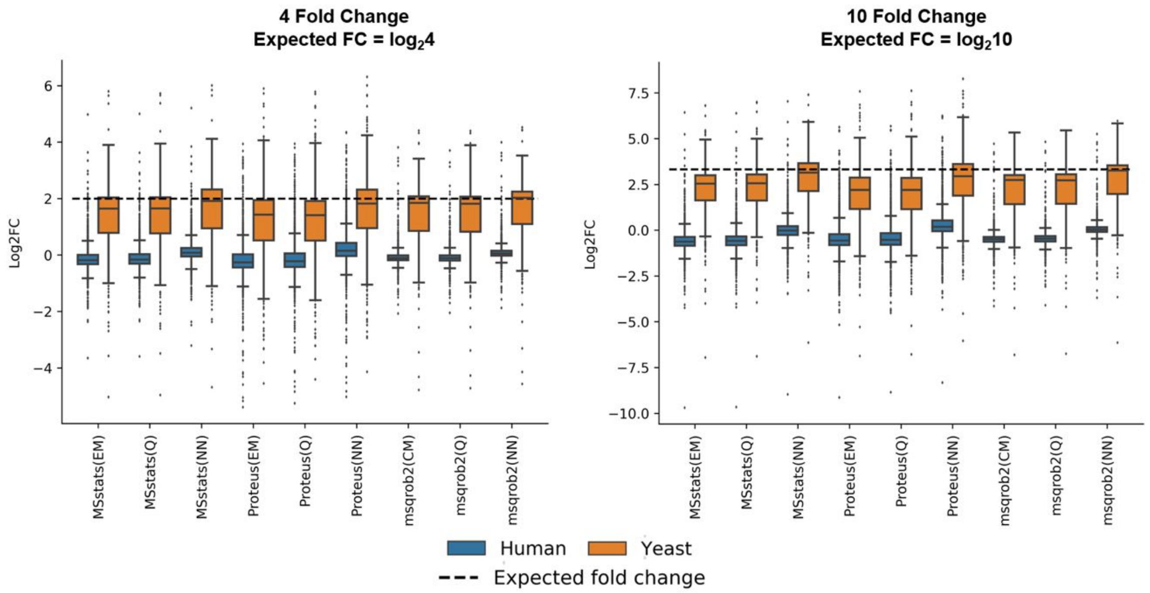

Supplementary Figure 5: Comparison of DE analysis on the PXD007145 dataset. The plot shows the number of differentially expressed proteins (DEP) that are returned by each tool at a certain FDR level, in which only the shared yeast proteins were assessed. The two vertical black lines indicate the 1 and 5% FDR levels.

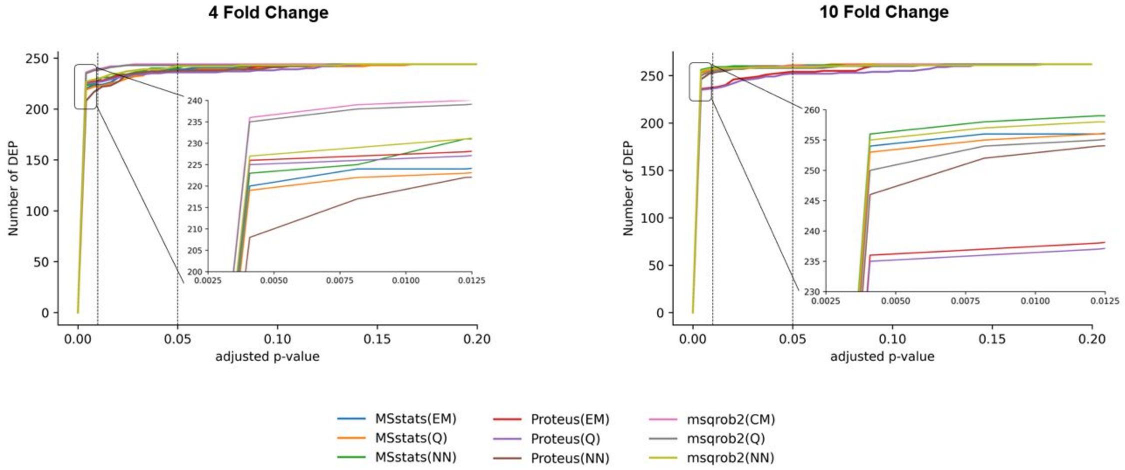

Supplementary Figure 6: Coefficient of variation for MSstats, Proteus and msqrob2 with different normalization methods for the peptide level analysis of PXD020248 with quantms.

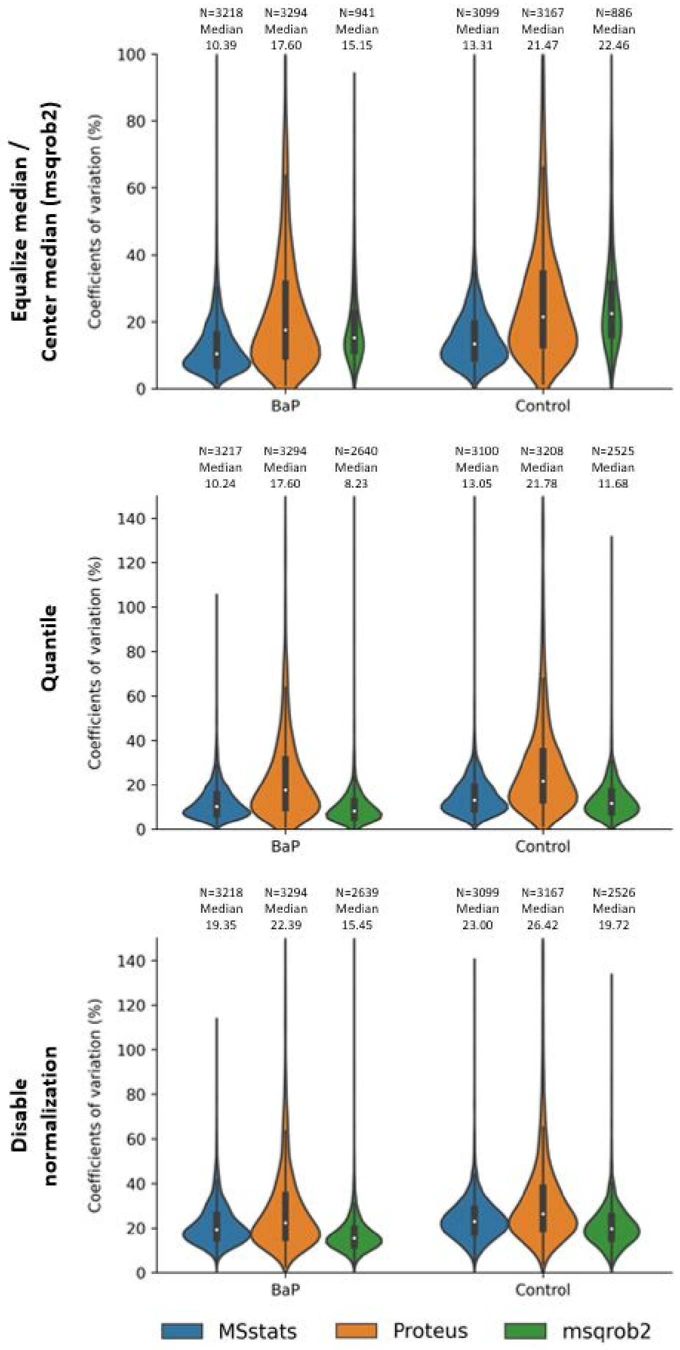

Supplementary Figure 7: Heatmap quality control reports for datasets PXD007145, and PXD020248, made by quantms.

(A) PXD007145

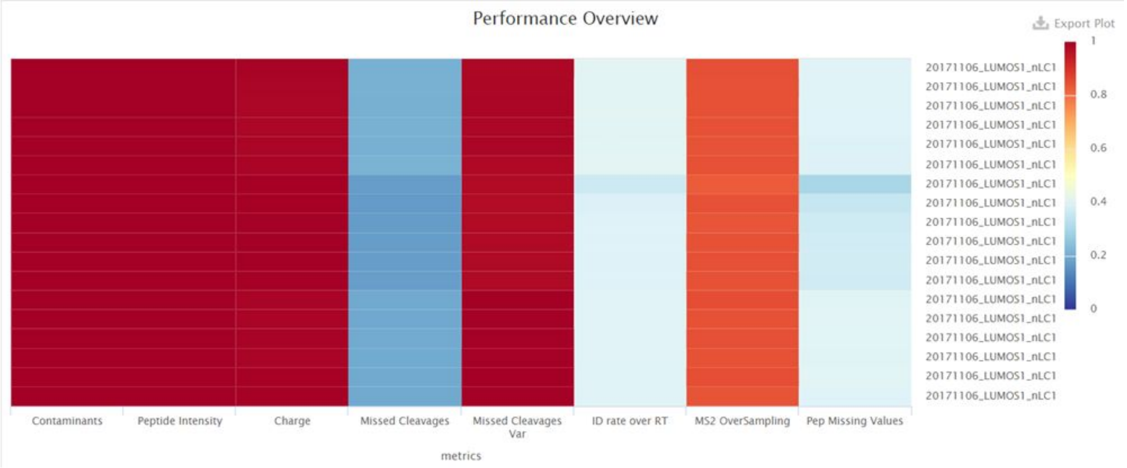

(B) PXD020248

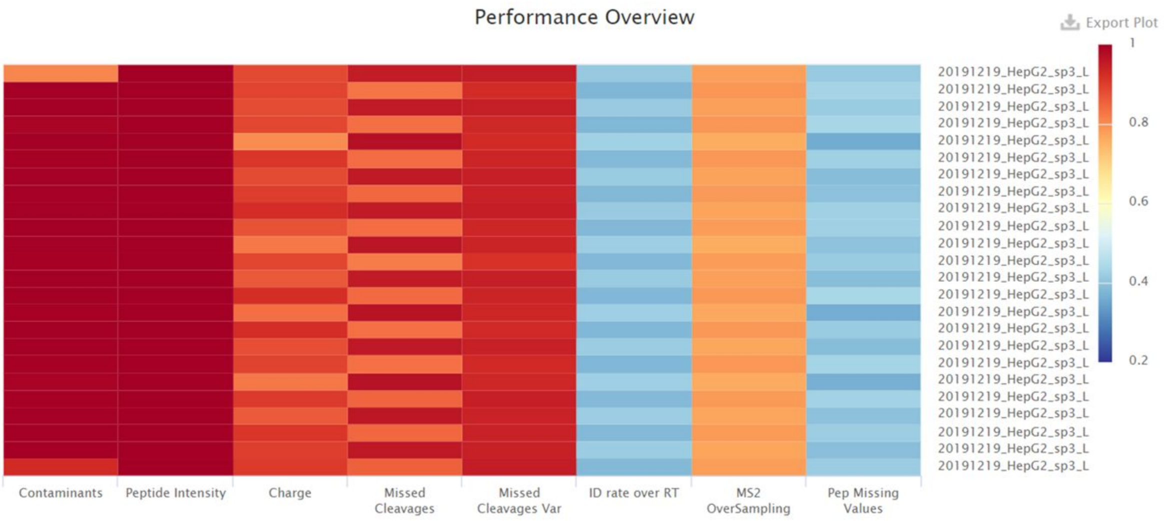

Supplementary Figure 8: The number of common DEP that returned at a certain p-value range for dataset PXD020248 analyzed with quantms.

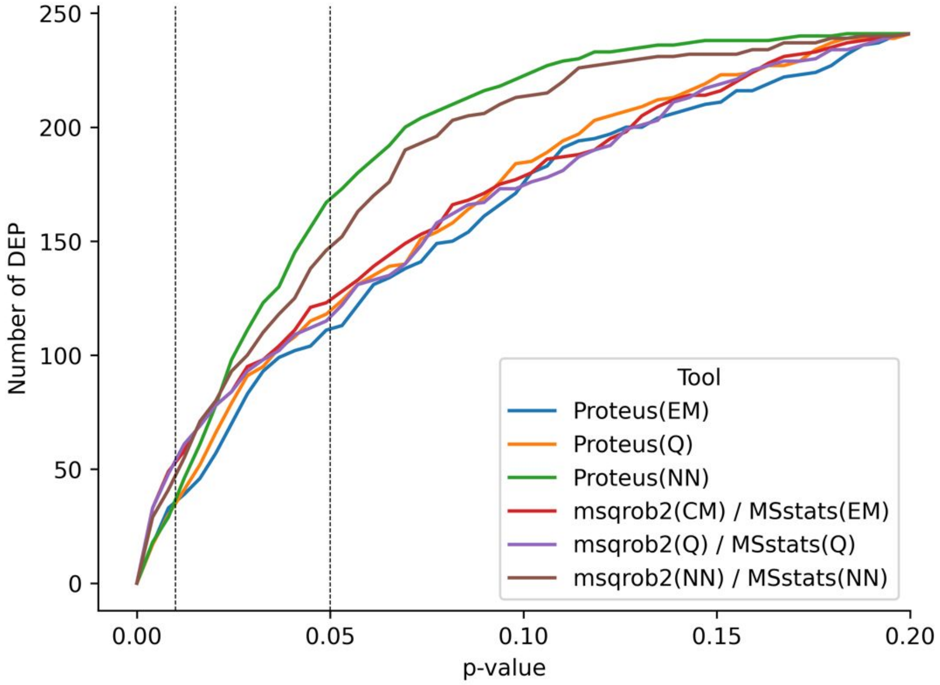

Supplementary Figure 9: Overlap of DEP between MSstats, Proteus and original results for dataset PXD020248 analyzed with quantms.

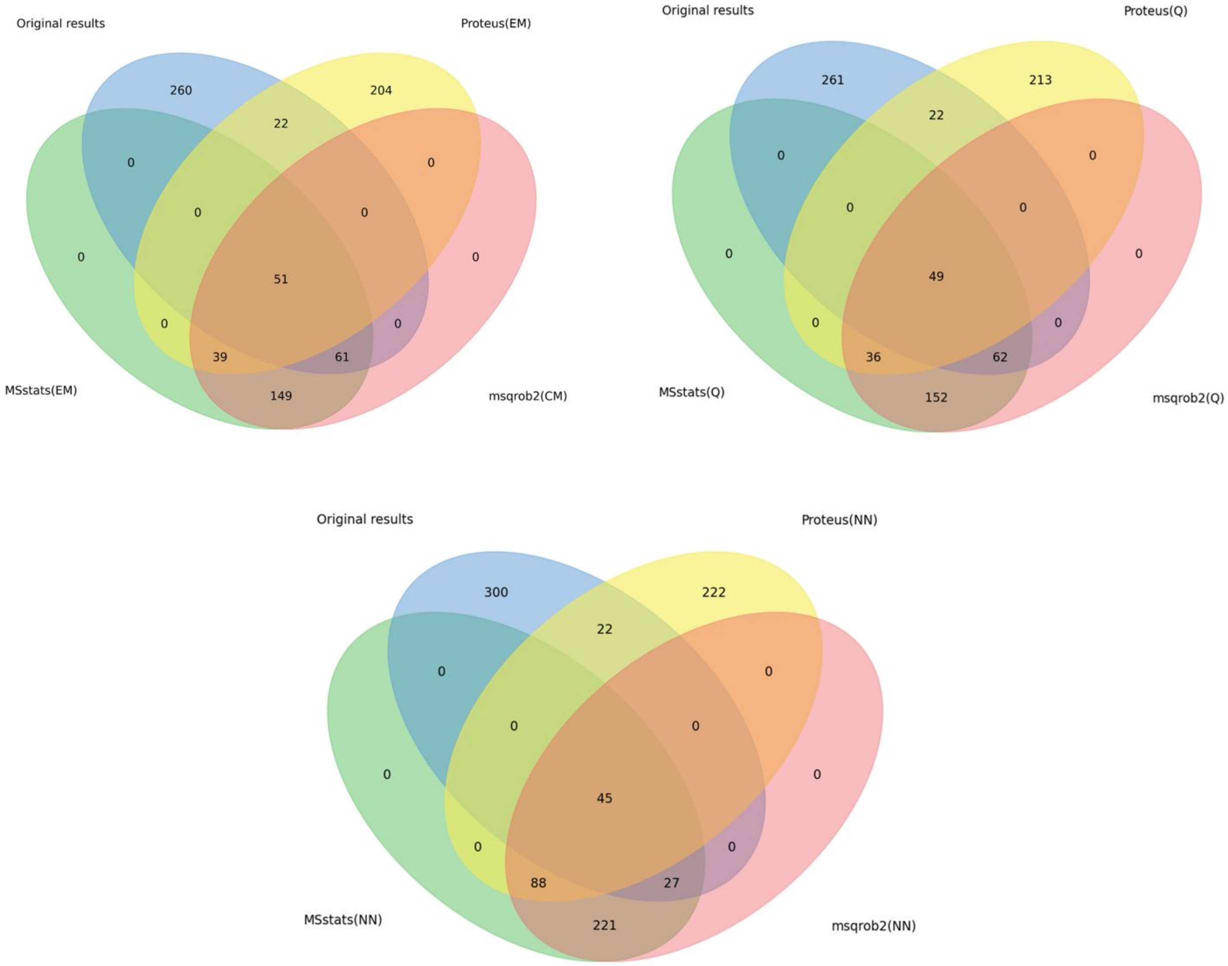

Supplementary Note 1: The operation record of the Shiny tools.

- **ProVision**

- All options are the default and have not been changed.
  - Minimum unique peptides: 2
  - Log2 transform data
  - Filter valid values: In at least one group
  - Impute missing values: Select width = 0.3, Select downshift = 1.8
  - Choose significance cut off: Less than 0.05
  - Choose log Fold change cut off: 1
  - Choose p-value adjustment: Benjamini-Hochberg FDR
- In the [Assign groups] option of the [Data handling] tab, modify the [axisLabels] column (refer to the [condition] column in "meta.csv" for the various datasets on the GitHub website).

- **LFQ-Analyst**
  - All options are the default and have not been changed.
    - Adjusted p-value cutoff: 0.05
    - Log2 fold change cutoff: 1
    - Imputation type: Perseus-type
    - Type of FDR correction: Benjamini Hochberg

- **Eatomics**
  - All options are the default and have not been changed.
    - Imputation method: perseus-like
    - Adjusted P value threshold: 0.05
    - Log Fold Change: 0 (0 here is to download all data, and the filter in the subsequent analysis is still 0.05)
  - In the [Select the clinical grouping factor] option of the [Differential Abundance] tab, choose [condition] option.

- **ProStaR**
  - The options that are not described are default.
  1. [Data manager] tab
     1 - Select file
       - Software to import from: maxquant
       - Is it a peptide or protein dataset: protein dataset
     2 - Data id
       - ID definition: Protein_IDs
     3 - Exp. and feat. data
       - Quantitative data: Choose [Intensity XX] data
     4 - Samples metadata
       - modify the [Condition] column (refer to the [condition] column in "meta.csv" for the various datasets on the GitHub website).
       - Choose the type of experimental design and complete it accordingly: Flat design (automatic)
  2. [Data processing (protein)] tab - [Filter data] subtab

In this part, only use [String-based filtering] function

- Column name: [Only_identified_by_site], [Reverse] and [Potential_contaminant]

3. [Data processing (protein)] tab - [Normalization] subtab
    - Normalization method: Global quantile alignment
4. [Data processing (protein)] tab - [Miss. values imputation] subtab

   [Partially Observed Values] function
    - Algorithm for POV: Det quantile

   [Missing on Entire Condition] function
    - Algorithm for MEC: Det quantile
5. [Data processing (protein)] tab - [Hypothesis testing] subtab
    - Contrast: One vs One
    - Statistical test: Limma
    - log(FC) threshold: 1
6. [Data mining] tab - [Diferential analysis] subtab

   [Pairwise comparison] function
    - Select a comparison: Choose the one we need

   [P-value calibration] function
    - Calibration method: Benjamini-Hochberg

   [FDR] function
    - p-val cutoff: 0.05
